# Revised FDI criteria for evaluating direct and indirect dental restorations—recommendations for its clinical use, interpretation, and reporting

**DOI:** 10.1007/s00784-022-04814-1

**Published:** 2022-12-12

**Authors:** Reinhard Hickel, Sabine Mesinger, Niek Opdam, Bas Loomans, Roland Frankenberger, Milena Cadenaro, John Burgess, Arnd Peschke, Siegward D. Heintze, Jan Kühnisch

**Affiliations:** 1 Department of Conservative Dentistry and Periodontology, University Hospital, Ludwig-Maximilians-University of Munich, Munich, Germany; 2 Department of Dentistry, Radboud Institute for Health Sciences, Radboud University Medical Center, Nijmegen, The Netherlands; 3 Department of Operative Dentistry, Endodontics, and Pediatric Dentistry Medical Center for Dentistry, University Medical Center Giessen and Marburg, Campus Marburg, Marburg, Germany; 4 Department of Medical Sciences, University of Trieste, Trieste, Italy and Children’s Hospital “Burlo Garofolo,” Institute for Maternal and Child Health, Trieste, Italy; 5 Louisiana State University Health Sciences Center, New Orleans, LA USA; 6 IvoclarVivadent AG, Research & Development, Schaan, Liechtenstein

**Keywords:** Dental restoration, Dental filling, Crown, Clinical assessment, Failure, Wear, Repair, Calibration

## Abstract

**Objectives:**

The FDI criteria for the evaluation of direct and indirect dental restorations were first published in 2007 and updated in 2010. Meanwhile, their scientific use increased steadily, but several questions from users justified some clarification and improvement of the living document.

**Materials and methods:**

An expert panel (*N* = 10) initiated the revision and consensus process that included a kick-off workshop and multiple online meetings by using the Delphi method. During and after each round of discussion, all opinions were collected, and the aggregated summary was presented to the experts aiming to adjust the wording of the criteria as precisely as possible. Finally, the expert panel agreed on the revision.

**Results:**

Some categories were redefined, ambiguities were cleared, and the descriptions of all scores were harmonized to cross-link different clinical situations with possible management strategies: reviewing/monitoring (score 1–4), refurbishment/reseal (score 3), repair (score 4), and replacement (score 5). Functional properties (domain F: fracture of material and retention, marginal adaptation, proximal contact, form and contour, occlusion and wear) were now placed at the beginning followed by biological (domain B: caries at restoration margin, hard tissue defects, postoperative hypersensitivity) and aesthetic characteristics (domain A: surface luster and texture, marginal staining, color match).

**Conclusion:**

The most frequently used eleven categories of the FDI criteria set were revised for better understanding and handling.

**Clinical relevance:**

The improved description and structuring of the criteria may help to standardize the evaluation of direct and indirect restorations and may enhance their acceptance by researchers, teachers, and dental practitioners.

**Supplementary Information:**

The online version contains supplementary material available at 10.1007/s00784-022-04814-1.

## Introduction

In 2007, an international workgroup published new FDI criteria [1–5] to evaluate the quality of direct and indirect restorations; an update with clinical cases was published in 2010. This diagnostic system classified aesthetic, functional, and biological properties and covers various types of failures (Table 1) by using 16 different categories [4, 5] with five grades for each criterion. In detail, scores 1 to 3 indicated clinically acceptable restorations, and scores 4 and 5 summarized clinically unacceptable situations indicating repair (score 4) or replacement (score 5). The criteria were approved by the Science Committee of the FDI World Dental Federation (FDI) in 2007 and the General Assembly in 2008 as standard criteria that were specially designed for use in clinical studies [1–5]. The authors outlined the potential of the criteria to be applied 1) in evaluations of new restorative materials or operative techniques in clinical trials, 2) for quality assessment of dental restorations in daily dental practice (mainly in simplified form), and 3) under- and postgraduate education to determine whether a restoration needs reviewing, refurbishment, reseal, repair, or replacement [6] (Table 2). A recently published review [7] indicated a growing use of the FDI criteria in clinical trials, which increased from 4.5% in 2010 to 50.0% in 2016. In addition to this positive trend, it needs to be recognized that the criteria set was also assessed as complex with a lack of consistency in some parts [7] and several questions from users indicated the need for clarification. Aiming at increasing internal validity and promoting widespread dissemination for scientific, practical, and educational purposes, the expert group decided to review and revise the previously published FDI criteria set to improve the clinical usability, practicability, and acceptability. Beside the clarification of ambiguous issues, it was aimed to specify the recommendations for its interpretation and reporting.

**Table 1:** Common material-dependent failures

Dental material	Common defects and failure patterns
(Resin modified) Glass ionomer cements	Cracks, chipping, bulk fracture, complete loose or lost restoration, or excessive wear
Composite and others	Cracks, chipping, bulk fracture, loose restoration (debonding), or complete loss of the restoration
Porcelain fused to metal	Chipping or delamination of the veneering layer, loose restoration (decementation/debonding), or complete loss of the restoration
All ceramic	Cracks, chipping, bulk fracture, loose restoration (decementation/debonding), or complete loss of the restoration
(Non)precious dental alloys	Perforation, loose restoration (decementation), or complete loss of the restoration
Amalgam	Cracks, creep, bulk fracture, or loose restoration or complete loss of the restoration

**Table 2:** Terminology and definitions and description of commonly used terms

Term		Definition
Methodology	Minor/slight	The terms “minor” and “slight” indicate a small difference in comparison to an excellent restoration. Differences are detectable by visual means or with additional procedures, e.g., short air drying or gentle probing. It represents a fully sufficient clinical situation, which does not need any further intervention
	Distinct	The term “distinct” indicates a clinically relevant difference in comparison to an excellent or good restoration. Otherwise, the clinical situation is basically acceptable and sufficient. Intervention by refurbishment potentially improves functionality or aesthetics
	Severe	The term “severe” indicates a substantial deviation in comparison to a sufficient restoration and characterizes a serious clinical condition which most likely requires operative intervention by repair or replacement
	Localized	Minor parts: less than half of the restoration (margin) is affected
	Generalized	Major parts: more than half of the restoration (margin) is affected
	Speaking distance	Typically ~ 80–100 cm/ ~ 3 ft. Dental operation light is switched off
	Examination distance	Typically ~ 40 cm/ ~ 1–1½ ft. The patient is placed on a dental chair, and the oral cavity is professionally illuminated. Tooth cleaning and short air drying of the teeth and restorations improve visual examination
	Tooth cleaning and air drying	A good examination of dental restorations requires the removal of the dental biofilm and tooth drying with compressed air for a few seconds until all saliva is removed. Avoid over drying!
	Visual examination	Visual examination without any magnification is the standard procedure for the evaluation of dental restorations. In case that magnifying loups or microscopes are used it needs to be reported. Acuity of operators and examiners should be regularly checked
Restorations defects	(Marginal) gap	Defective interface between the dental hard tissue and the restoration material, which is leaving parts of the restoration margin clinically exposed. A wide range of width and depth is possible. Optimally, there is a smooth transition between the dental hard tissue and the restorative material
	Negative/positive step	Steps are differences in height between the dental hard tissue and the restorative material. A step is formed due to under-contour (negative step) or over-contour of the restoration at the restoration margin (positive step). Different dimensions are possible
	Enamel and dentin cracks/cracked dental hard tissue	Crack lines in enamel/dental hard tissue are commonly detectable in (un)restored teeth and mostly represent no pathology. Nontraumatic tooth cracks have a wide clinical spectrum and reach from small enamel breakdowns to complete tooth fractures. If such a clinical situation directly involves a restoration or its margin it will be considered in the category “Dental hard tissue defect at restoration margin (B2)”. Traumatic dental injuries have to be separated from this entity
	Material crack	Crack lines within the restoration material may indicate that restoration could not withstand occlusal forces and might be interpreted as an initial material fracture
	Fracture	There is a huge spectrum, which reaches from small defects (chipping fractures) to a substantial loss of material (bulk fractures). Typically, a residual restoration material is present and cavity walls are exposed
	Bulk fracture	Fracture within the body of the restoration mostly perpendicular to the occlusal surface
	Chipping/Chip fracture	A chipping is a minor or major cohesive fracture of tooth-coloured restoration material or an indirect restoration with a veneered framework mostly parallel to the occlusal surface. In most cases the overall functionality of the restoration is not affected and the chipped area can be polished or repaired
	Delamination	Partial or complete adhesive failure of the veneering material of an indirect restoration
	Decementation	Loose or lost conventionally cemented indirect restoration. Typically, loose/lost but proper indirect restorations can be recemented/reluted
	Debonding	Loose or lost adhesively bonded direct or indirect restoration. Typically, loose/lost direct restorations have to be replaced. Loose/lost but proper indirect restorations can be recemented/reluted (= repair)
	Loss of retention	A restoration can be fully retained, partially retained or lost. Furthermore, each type of restoration can be adapted to the dental hard tissue (full retention) or decemented/debonded (loss of retention). Loose or lost, but properindirect restorations can be recemented/reluted. Loose or lost direct restorations have to be replaced
	Caries at restoration margin (CAR)	CAR is located directly at the restoration margin without sound tooth structure in between. CAR can reach from a non-cavitated carious lesion to large cavities. It represents a new carious process at the restoration margin. Demineralisations can be left at cavity margins during restoration placement as part of a minimal invasive intervention strategy
Intervention/management strategies	5R	The “5 Rs” include reviewing/monitoring, refurbishment, resealing, repair, and replacement of deteriorating or failed restorations [6]
	Reviewing	Regular monitoring in risk-related and individualized intervals
	Refurbishment	Refurbishment is a minimal invasive, subtractive intervention, which includes contouring of the form and/or margins as well as polishing of the restoration`s surfaces to reduce biofilm accumulation. No new adhesive, sealant, or filling material will be added
	Reseal	Reseal/sealing is a noninvasive, additive technique, which includes the direct application of an adhesive or sealant on gaps or defects without cavity preparation. Typically, superficial localized marginal gaps can be sealed
	Repair	Repair is a minimal invasive, additive technique that involves the direct application of restorative material after minor cavity preparation or roughening/conditioning of remaining surfaces (artificial/biological surfaces) and preservation of sufficient parts of the existing restoration. Typically, localized defects with clinical access can be repaired, e.g. chipping, minor bulk or cusp fractures or CAR
	Replacement	Replacement is required if the restoration defects are so extensive that a repair is not reasonable. This procedure requires the removal of the existing material, cavity/tooth preparation and the application of a new direct or indirect restoration

## Materials and methods

The existing FDI criteria [4, 5] have been improved by using a structured process to obtain information from a group of experts by means of a series of meetings and/or evaluations. This process with multiple rounds of feedback, open discussion, and rephrasing was iteratively continued until no further changes in the documents were needed [8]. A group consensus process is crucial in building guidance recommendations [9, 10]. In detail, the present information flow included a systematic search of the literature, a kick-off workshop under the participation of all experts as well as a structured communication flow aiming to converge existing opinions, and, finally, to reach a unanimous group consensus about the revised clinical criteria for the evaluation of direct and indirect dental restorations.

### Expert panel

Ten experts in conservative and restorative dentistry agreed to participate, discuss, revise, and rephrase the criteria in spring 2019. Three of those (RH, SH, and AP) were also part of the original expert team. As several colleagues from previous projects [4, 5] were not available anymore due to different reasons, the work group was re-formed aiming at including experts from restorative dentistry of different regions.

J. Kühnisch and S. Mesinger coordinated the Delphi method as facilitators and collected all responses from the experts from the beginning, analyzed the opinions, structured the information, identified conflicting viewpoints; furthermore, they revised all documents accordingly. R. Hickel acted as a moderator during the workshops, online meetings, and discussions; furthermore, he provided numerous questions, comments, and suggestions by scientists, which he collected after the initial publications. Participants were forced to freely and consistently express their opinions and were encouraged to provide criticism or feedback and to detect errors or conflicting viewpoints. Although a consensus process captures collective knowledge, it should be noted that such criteria set may be, to some degree, a subjective viewpoint of the expert group [11].

### Delphi method

The Delphi process was initiated with a group workshop at the Department of Conservative Dentistry and Periodontology in Munich, Germany, on June 3–4, 2019. During this face-to-face meeting, the existing scientific literature was presented and critically discussed, and empirical experiences of the existing scoring criteria for direct and indirect dental restorations were reviewed. In addition, a preliminary draft of a revised FDI criteria set was proposed based on the latest version [4, 5]. As a result of the workshop, the need and methodology for improvement were justified and agreed upon. After the meeting, the initially revised FDI criteria set was distributed, evaluated, and consistently updated. The following group discussion was held during an online meeting on September 16, 2019. The main intention of this meeting was to agree on the simplified structure and the importance of each category. This process continued until spring 2020, and the resulting criteria set was then pre-tested by the expert panel in a reproducibility study using intraoral photographs of different restorations with a broad spectrum of deficiencies. This study was performed in two rounds from May to July 2020. Feedback from the experts and statistical analyses of the intra- and inter-examiner reproducibility were compiled and discussed during other online meetings (July 21, 2020 and September 21, 2020). Further, where some inconsistencies or ambiguities were remarked, minor modifications were made to the FDI criteria set to harmonize the scores in each category. Diagnostic evaluations were repeated in a third round using the above set of clinical images. The final version of the revised FDI criteria was reviewed again by the whole expert panel and unanimously agreed on during another web meeting on November 9, 2020. The results of the reliability study were summarized in a separate report [12].

## General considerations for clinical studies on dental restorations

In restorative dentistry, it is mainly evaluated how the material or restoration responds to the oral cavity of the patient with factors that may influence the success of the restoration, such as chewing forces, bruxism, diet, saliva, and the oral biofilm. Therefore, there are many confounders like patient factors, e.g., age, gender, tooth substance, chewing forces, oral hygiene, chewing tobacco, diet, general diseases, and local biological factors, e.g., location in the mouth, caries risk and periodontitis risk, and operator factors, e.g., clinical experience, decision making, and skills, which all influence the clinical performance of a dental restoration.

### Study type and design

Several study types require a quality assessment of dental restorations. Here, clinical studies on new materials have to be mentioned primarily, which typically need a comprehensive restoration assessment after placement, during follow-ups, and at the final examination visit. Three- and 5-year follow-ups are at least advised for direct and indirect restorations, respectively. Longer observation periods are recommended especially when a new type of treatment or material is to be evaluated. For an observation period of 3 years, up to five recall sessions might be helpful. Ideally, the baseline evaluation should be carried out approximately 1 week after the insertion of the restoration and not during the placement appointment. If this procedure is not possible, the assessment by different dentists in the same appointment and an audio call interview 1 week later would be an acceptable compromise. Aiming to increase trial efficiency, baseline evaluation might also be performed after tooth rehydration approximately 30–60 min postoperatively and by checking the functionality no later than 4 weeks. The remaining recalls can be scheduled after (6), 12, 24, and 36 months. For longer observation periods, (bi)annual recalls might be preferable.

Furthermore, the quality of restorations could be evaluated in practice-based, epidemiological, observational, or diagnostic studies. In daily practice routines, practitioners are consistently forced to evaluate different aspects of restored teeth, which should be done with a validated and widely accepted set of criteria. When considering the whole spectrum of study types, it is understandable that the choice of categories and grades depends on each study’s intended purpose and methodological requirements. For clinical trials, the preferable examination setting is a dental unit with compressed air and standard illumination. Additional magnification tools, e.g., magnifying loupes, or documentation methods, e.g., intraoral photographs or 3D scans, may accompany visual examination. For practice-based studies, a simplified methodology might be more relevant. However, reporting of all chosen procedures is essential to better compare studies and interpret the results adequately.

### Study population

It is recommended that clinical studies be conducted on the intended target population according to predefined and rigorously applied patient- and tooth-based inclusion and exclusion criteria, e.g., age range, gender, ethnicity, caries experience/risk or activity (high vs. low), parafunction or bruxism (present or not present), temporomandibular disorders (TMDs), oral hygiene (good, moderate, bad), smoking/vaping habits (no, moderate, heavy), or diet habits, e.g., coffee, tea, soft drinks, acidic foods, and beverages. Other habits of patients, such as frequent use of chewing tobacco or bubble gum, or parafunctions such as nail and/or thumb chewing, may also potentially influence the longevity of restorations and therefore need to be reported and re-evaluated with respect to the inclusion and exclusion criteria on each follow-up examination.

In addition to patient-related factors, it is essential to consider tooth-related variables. Here, the type of dentition (primary, mixed, permanent), tooth type (anterior, premolar, molar), quadrant, and affected surfaces are relevant. Furthermore, Black’s cavity class, the location of the cavity margin in relation to the gingiva (supra-, equi-, subgingival), and the hard tissues involved (enamel vs. dentin), the caries excavation technique and endpoint (selective vs. complete caries removal), as well as the type of antagonist teeth (unrestored vs. restored tooth, restoration material, not present) may be clinically relevant. Importantly, the indication to (re)place a restoration should be justified strictly according to common dental pathologies: 1) primary caries (proximal, occlusal, cervical, root, early childhood caries), 2) non-carious hard tissue defects, e.g., erosive tooth wear, abrasion, fractures/cracks or trauma, 3) dental developmental disorders, e.g., molar-incisor-hypomineralization or hereditary disorders of enamel/dentin, and/or 4) other specific situations, e.g., restorations to improve aesthetics due to discoloration or diastemas. The pooling of restorations with different characteristics in one clinical study, e.g., classes I and II, anterior and posterior teeth, or carious indications, e.g., caries and developmental disorders, should no longer be an accepted procedure. The flow of screened, eligible, and finally recruited patients/restorations should be described and illustrated as a flow chart according to the relevant reporting guideline for each study type, e.g., the CONSORT statement for randomized controlled trials [13, 14]. Beside this, patient’s motivation to adhere to the study protocol should to be safeguarded. Here, information cards might be helpful to provide data for the patient and dental professionals.

### Evaluation of dental restorations

The quality assessment of dental restorations is a stepwise decision-making process that includes, if needed, the following procedures: 1) professional tooth cleaning and short air drying of the restored tooth for a few seconds, 2) functionality checks with standardized probes and blades, 3) static and dynamic occlusion testing with articulation paper, and 4) cold stimulus aiming at assessing hypersensitivity and pulpal reactions. It is also important to understand that the number of included categories can be chosen flexible according to the study aim and design. Furthermore, it can be decided if the scoring for each category will consist of five grades (excellent/good/satisfactory/unsatisfactory/poor) or, in a simplified form, only three grades (sufficient/acceptable = score 1 to 3, insufficient/inacceptable but repair possible = score 4, and insufficient/inacceptable but repair not possible/reasonable = score 5). The latter approach might be of relevance especially in practice-based studies. As some of the earlier described 16 categories were rarely used in clinical trials [7], the revision includes only the most frequently used ones now. The categories for general health, gingival, periodontal, and mucosal conditions, erosive tooth wear, or abrasion [15–35] were separated from the “core” categories, as most of them are not directly related to the evaluation of dental restorations but reflect the status of the tissues beneath restored teeth (Table 3).

**Table 3:** Additional clinical parameters and the corresponding indices that might also be scored beneath dental restorations

Clinical parameter	Corresponding index and/or set of criteria
General health status	The ASA physical status classification system is a system for assessing the fitness of patients before surgery/treatment and was developed by the American Society of Anesthesiologists [15]
Allergy	Medical history and/or allergy testing
Tooth vitality and pulp pathology	Pain anamnesis, sensibility test on cold, percussion test, pain on palpation, pain on chewing
Surface staining	Black stain, food-associated staining (coffee, tea, tobacco, and others)
Plaque accumulation and calculus	Quigly Hein index [16], plaque index [17, 18], and others
Gingival health	Gingivial index [19, 20], sulcus bleeding index [20], modified sulcus bleeding index [21], papillary bleeding index, bleeding on probing or brushing, and others
Periodontal health	Classification scheme for periodontal and peri-implant diseases and conditions [22] and others, community periodontal index CPITN index [23], measurement of attachment loss and pocket depth, bleeding on probing [24], and others
Mucosa pathology	Potentially malignant disorders of the oral mucosa and oral epithelial dysplasia [25]
Caries	DMF index [26], ICDAS [27], UniViSS [28, 29]
Erosive tooth wear	Basic erosive wear examination [30]
Attrition and abrasion (tooth wear)	Tooth Wear Index [31]
Hard tissue fractures and cracks	Tooth cracks[32]
Developmental defects	Molar-incisor hypomineralization [33], fluorosis index [e.g., 34], and others
Dental trauma	Crown fractures [35]

### Training and calibration

Clinical assessments in studies should always be carried out by trained and calibrated examiners. Therefore, appropriate theoretical and practical training sessions are mandatory and guarantee the consistency of judgments throughout the whole study period. Furthermore, the documentation of training is crucial. Each trainee should have a similar reproducibility rate in comparison to the trainer, which can be statistically expressed as intra- and inter-examiner reproducibility [36]. Clinical examples were published to assist study groups with this exercise [4, 5] and the revised FDI criteria set can be downloaded as illustrated document from the journal website. Nevertheless, calibration on patients in a clinical setting cannot be replaced by the evaluation of photographs, but time-consuming clinical calibration sessions might be shortened.

### Recommended statistics

Studies on restoration quality and longevity require observations over a time, where different events, e.g., loss of patients, loss of teeth due to (non)study-related reasons, or failure of test restorations can occur. This implies an appropriate follow-up process and documentation of subjects, restorations, and failures. It is suggested to provide absolute numbers of failures and the overall number of evaluated restorations for each examination time point. There are different ways to calculate the *mean annual failure rate* or the normalized failure index besides the simple one dividing the total failure rate by the number of observation years [e.g., 37, 38]:

$$mean\;annual\;failure\;rate\;\left(mAFR\right)^1=1-\sqrt[t]{1-(\frac{N_{\mathrm{Failures}}}{N_{\mathrm{Restorations}}})}$$

$$mean\;annual\;failure\;rate\;\left(mAFR\right)^2=-\log(1-(\frac{N_{\mathrm{Failures}}}{N_{\mathrm{Restorations}}}))/\mathrm t$$

$$Normalized\;Failure\;Index\;(NFI)=\frac{N_{\mathrm{Failures}}}{(N_{\mathrm{Restorations}}\ast t)}$$

*N*_Failures_: total number of failed restorations
*N*_Restorations_: total number of investigated restorations
*t*: observation time
^1^: preference of this formula in case of low failure rates
^2^: preference of this formula in case of high (almost 100%) failure rates in less than 1 year.

For calculating of the *success rate*, the dichotomization of the data into sufficient (scores 1–3) and insufficient (scores 4 and 5) is needed. The calculation of the *survival rate* uses the dichotomization of the data into restoration present including repaired (scores 1–4) and not present/failed (score 5). Kaplan–Meier curves are frequently applied to illustrate the success or survival probability over time [36]. The log-rank test is usable to compare differences between groups [39–41]. In addition, Bonferroni corrections or multivariate analyses, e.g., Cox proportional hazards model or Poisson distribution can be computed. When considering the potential influence of all patient-related factors on restoration survival, it is recommended to conduct a multiple logistic regression analysis.

Following the aim of increasing the internal validity domains and categories is somewhat rearranged in relation to their clinical relevance and importance; therefore, the functional properties (domain F) were now placed at the beginning of the assessment followed by the biological (domain B) and the aesthetic properties (domain A). The revised FDI core criteria set summarizes 11 criteria. In addition, the criteria “patient’s view” and “radiographic evaluation” were shifted in the new domain “miscellaneous” (domain M).

## Domain F: functional properties

The assessment of the function of a restoration is a key issue in scientific studies as well as in daily dental practice. Here, the visual examination provides relevant information that is sometimes hard to objectify and quantify. Therefore, the use of metric instruments improves the validity of the criteria. For this purpose, standardized instruments, e.g., metric probes and blades, are recommended to use the FDI criteria reliably.

### Fracture of material and retention (category F1)

Restoration fracture and retention are the most relevant categories in clinical practice when evaluating direct and indirect restorations and therefore should be included in any study. Different fracture patterns and retention failures may occur in relation to the type of restoration: cracks, chipping/delamination, bulk fractures, or incomplete and complete loss of retention (Table 1). Minor material chipping or hairline cracks, which sometimes can only be detected after tooth cleaning and air drying, most often do not require an operational intervention, but these events should be monitored in follow-up visits recorded during data capture. Small chipping fractures with loss of material might only be monitored or corrected by refurbishment, e.g., recontouring and polishing. The main reason for failures of direct composite restorations is bulk fractures [41, 42], which can potentially be repaired. In cases of severe or multiple bulk fractures, replacement of direct restorations is considered the treatment of choice [41–44]. Types of fracture patterns are sometimes different in indirect restorations (Table 1). Material chipping of variable extension is quite common in veneered ceramic restoration. In monolithic ceramic restorations, bulk fractures are more common. Ceramic fixed partial dentures primarily fracture in the connector area [45, 46]. Bulk fractures or delamination of a greater volume may substantially affect restoration integrity. If the loss of material is localized, repair might be possible. Repair of a restoration with extended or multiple fractures might not be reasonable, and complete replacement is more appropriate.

Severe loss of retention is in any case insufficient (scores 4 and 5), but the extent of lost material, partially or (almost) completely, defines whether a direct restoration might be repaired or not. If a restoration is graded as completely loose or lost (score 5), all other functional and aesthetic categories usually become not applicable. Indirect restorations, which can be recemented/reluted, will be rated with score 4 (repair).

### Marginal adaptation (category F2)

There are different interfaces between the dental hard tissue, restorative material, and adhesive and/or luting resin/cement layer. Each interface can degrade and potentially alter marginal adaptation. In clinical practice, it is impossible to distinguish failures between the different interfaces. Therefore, only the marginal adaptation as such can be assessed. The quality of marginal adaptation is both the result of the properties of the adhesive, luting resin/cement, and restorative material and the skill and knowledge of the operator to create a good restoration (adequate cavity preparation, moisture control, application of materials according to instructions for use) [1–5, 41].

Evaluation of marginal adaptation should be done by visual examination and the use of a metric 250-µm probe, e.g., Fissuren Sonde 250EX with 250 µm diameter (Deppeler, Rolle, Switzerland). With respect to practicability, another probe with a diameter of 150 µm is no longer preferred. Ideal marginal adaptation shows a smooth transition from the restoration material to the surrounding tooth structure; no marginal irregularities should be detectable by gentle probing. Minor marginal deficiencies can be detected as discoloured margins or ditches and will be categorized as “sufficient” [47]. Wide (> 250 µm) marginal gaps with a gap depth ≥ 2 mm indicate a situation of clinical insufficiency and probably require dental intervention depending on both the location and the caries risk/activity/history of the patient [4, 5, 48–50].

### Proximal contact point (category F3)

The tightness of proximal contact points should be estimated in a reproducible manner. Metal blades (e.g., matrix for EX kit; blades’ thickness 0.025 0.05, and 0.1 mm; Deppeler, Rolle, Switzerland) are recommended for better categorization [1–5, 51]. In case of unavailability of the blades, waxed dental floss might be considered a non-standardized alternative. A proximal contact point has a physiological strength when the 25-μm metal blade (or dental floss) can pass through it with resistance [1–5]. An appropriate degree of contact strength as well as a properly located contact area is recommended to prevent food impaction and allow for interdental papilla to fill the interproximal space [52, 53]. The lack of physiological contact point strength may result in food impaction, papillitis, or discomfort. However, it must be addressed that physiological contact strength can vary considerably among patients [54]; therefore, the strength of proximal contact points should be assessed individually and with caution. For better evaluation, an adjacent contact for comparison could be used. In addition to less optimally restored contact areas, weak or no contact points could be linked to the individual tooth form, e.g., microdens, atypical tooth position, diastema, and/or paced/gap-toothed dentition. In these clinical situations, this criterion shall not be applied. The same applies to patients with advanced periodontitis or mobile, flared, or missing teeth. Generally, teeth with nonexisting proximal contacts cannot be evaluated with regard to proximal contact points.

An unintentionally interlocked contact point due to excessive restorative material, bonding agent, luting resin, or cement, which makes it impossible for a blade or dental floss to pass, has been added to the revised FDI criteria set. Unintentionally interlocked contact points are unacceptable, as they impede oral hygiene, make affected tooth surfaces inaccessible for proper cleaning, and may therefore cause caries and/or periodontitis.

### Form and contour (category F4)

In modification to the previously published recommendations [1–5], this category is now listed under “functional properties,” because both are essential variables of the physiological functionality of any restoration in the masticatory system [55]. As characteristics of an optimally restored tooth form and contour, the following indicators of functionality have to be mentioned: (1) gingiva and periodontium are protected, (2) physiological embrasures between teeth are rebuilt and potentially allow for an alignment of the interdental papillae [52], (3) a spillway for the passage of food during mastication is reconstructed and prevents gingival food impaction, (4) the restoration safeguards the self-cleansing ability and the occlusal embrasure allows for better access for oral hygiene floss passage, and (5) stabilizes the position of the tooth to adjacent and antagonistic teeth. It needs to be further noted that optimal reconstruction of form and contour not only guarantees functionality but also substantially affects aesthetics. In anterior teeth, angulation and width-to-height ratio should be additionally considered [56].

The individual rebuilding of form and contour depends on the patients’ wishes as well as on the dentist’s or dental technician’s skills. An ideal form and contour may not be achievable in the case of children/adolescents or elderly individuals with reduced compliance, disabled patients, individuals with dental anxiety, or patients with limited mouth opening. Furthermore, irregular tooth angulation and/or position in the jaw, e.g., due to tooth crowding, may also complicate an ideal restoration in terms of form and contour. Irregularities, including overhangs and positive steps of the restoration, should be improved by refurbishing to avoid negative side effects, e.g., plaque accumulation and marginal discoloration. Underfilling might be the result of a primarily under-contoured restoration or/and of a gradual process of deterioration of the restorative material.

### Occlusion and wear (category F5)

The static and dynamic occlusion of a restored tooth influences the functionality of the dentition. An ideal occlusion of the restoration should be harmonized with the individual and age-related occlusion of the masticatory system. The restoration with antagonistic teeth should not have a non- or hyper-occlusion; it should avoid biomechanical stress on the supportive tissues and should not trigger pain and TMDs. A restoration with non-occlusion potentially limits the chewing ability and may result in the elongation/super eruption of the restored and/or the antagonist tooth if there is no contact to prevent this. Oversized cusps or dimensions of the occlusal surface could lead to premature contacts, hyper-occlusion, and interfering balances, which may negatively influence the restoration’s longevity in terms of material chips or fractures (category F1) and may cause discomfort, pain, or TMDs [57–63].

Wear is the result of dynamic processes predominantly on occlusal and proximal surfaces of the restoration, which might be influenced by individual occlusion, bruxism, individual habits, and nutritional/chemical and mechanical challenges. Wear can hardly be assessed by clinical examination alone; therefore, objective monitoring methods are required, which can directly compare follow-up with baseline information, e.g., plaster models or 3D scans [64]. Therefore, only a simplified recording in the revised FDI criteria set has been integrated. If quantitative information on wear is needed, intraoral 3D scans or scans of replicas after impression-taking should be considered as the method of choice [65–67].

## Domain B: biological properties

Pathological processes that are related to dental restorations include caries at the restoration margins (B1), dental hard tissue defects, cracks or fractures (B2), and postoperative hypersensitivity or pulpal inflammation (B3). Several other dental pathologies, e.g., developmental dental defects, bruxism, and erosive tooth wear, may potentially interfere with longevity. With respect to the aim of clarification and prioritization, the most frequent pathologies are covered in the revised criteria set. Therefore, particular research questions may require the inclusion of additional standard methods (Table 3).

### Caries at restoration margins (CAR, category B1)

This category has been harmonized in relation to the current caries definitions [28, 68–71]. Caries is the most prevalent dental disease from a global perspective [72–74], and the etiology of caries at restoration margins (CAR, synonyms: secondary caries or recurrent caries) is not different from that of primary caries [75–78]. CAR is mostly located in plaque stagnation niches, e.g., proximal margins, and can rarely be diagnosed on smooth surfaces that are well accessible for oral hygiene [79]. Furthermore, it may require significant gaps that are accessible to oral fluids in a caries active oral cavity to contribute to the risk for CAR. Some early clinical studies conclude to minimum gap width occlusally of 400 microns and 250 µm approximally [1, 2, 48], but recent in situ studies also show that gaps as small as 50 microns might be able to generate CAR [80, 81]. For margins at the proximal gingival box of a class II restoration, smaller gaps or even no gap maybe associated with caries at this site. The most important factor regarding CAR is the caries activity of the patient.

Clinically, CAR reaches from non-cavitated carious lesions to deep cavities. While initial carious lesions require no (reviewing, topical fluoride application) or minimally invasive intervention reseal or refurbishment, cavitated lesions at the restoration margin probably need operative dental measures in terms of repair or replacement depending on the size of the defect and restoration and caries activity of the patient [71, 82, 83]. Importantly, if any caries occurs at any other site of a tooth that is not *directly* related to the restoration, it should not be registered as CAR. The clinical diagnosis of CAR is sometimes difficult to differentiate from stained margins [76, 77]. Importantly, stained restoration margins with no demineralized hard tissue should not be confused with CAR.

### Dental hard tissue defects at the restoration margin (category B2)

This criterion comprises tooth cracks, enamel chipping, or cusp fractures at the restoration margin. Additionally, cracked tooth syndrome [84] is considered, which may also cause hypersensitivities or pain. In addition, it is noteworthy not to include other events in this category, such as physiological attrition and wear, abfraction, or defects related to other reasons, e.g., trauma. Additionally, a lost restoration material or CAR must be scored in their corresponding categories. Clinical assessment could be supported by light transillumination of the restored tooth.

### Postoperative hypersensitivity and pulpal status (category B3)

Postoperative hypersensitivity is linked to pulpal reactions immediately after placement of a dental restoration and can include discomfort, pain, pulpitis, or, later, loss of tooth vitality. There are several factors that affect the pulp-dentin complex: 1) diagnosis and history of the tooth, 2) dental treatment including cavity preparation, caries excavation, or placement of a properly sealed restoration, 3) properties of the adhesive, luting resin, and/or restoration material, and 4) the patient’s individual pain perception. In general, the diagnosis of postoperative hypersensitivity may indicate the presence of a deficiency during the restoration workflow, e.g., incomplete adhesive bonding of the restoration, which is probably difficult to identify later.

With respect to definition, tooth sensitivity needs to be recorded before and after restoration placement and at all recall visits. On each examination, it is necessary to consider, first, the patient’s reporting of tooth (hyper)sensitivity, e.g., by using a visual analogue scale, and second, testing of irritability of the pulpal nerve on cold, e.g., with dry ice or cold spray, in comparison to the reaction of a contralateral, sound, and unrestored tooth. The restoration should be rated as acceptable when normal sensitivity or mild pulpal symptoms are recorded during follow-up examination. In cases of postoperative hypersensitivities, transient pain or more intense pulpal reaction, individual monitoring intervals might be indicated. Irreversible pulpitis or pulp necrosis requires endodontic intervention to overcome the problem.

## Domain A: aesthetic properties

The aesthetic performance of dental restorations can be characterized by surface luster, surface texture, marginal staining, color match, and anatomical form. The evaluation is somewhat subjective and therefore more prone to potential bias and variability [85, 86]. The aesthetic appearance of a restoration depends mainly on how well it blends into the surrounding tooth structure, which is influenced by oral hygiene.

The evaluation of aesthetic properties is of clinical relevance for visible and tooth-colored restorations within the smile frame only, usually canine to canine. In many patients, the mesiobuccal aspect of upper premolars is visible when patients smile and therefore essential for aesthetic appearance. In most individuals, however, the evaluation of aesthetics in posterior teeth is less important. Depending on the study design, setting, and aim of the investigation, researchers can choose if the evaluation of the aesthetic properties should be evaluated from a standard examination distance under operating light (~ 40 cm) or from a speaking distance (~ 80–100 cm), which will lead to different results and should therefore be mentioned. Additional devices to objectify aesthetics are intraoral photographs or scans, color scales, colorimeters, spectrophotometers, or 3D imaging.

### Surface luster and surface texture (category A1)

Surface luster and texture are created by the reflection of light from the surface of the restoration, which mainly depends on material properties and the restoration surface [87]. In detail, the intrinsic material roughness (nano- and micro-roughness), finishing procedures (macro-roughness), e.g., polishing marks, and flaws due to material properties or material processing, e.g., pores and voids, must be considered. Macroscopic deviations in surface texture, such as polishing marks or pores, are easier to detect by visual examination than minor deficiencies [87, 88]. Ideally, the surface luster and texture of the restoration are comparable to that of the surrounding hard tissue.

### Marginal staining (category A2)

Marginal staining is defined as the discoloration of a crevice between the cavity wall and the restoration, subsequently affecting the margin of the restoration, which should not be confused with caries [76, 77]. A prerequisite for staining is the presence of a ditch or gap at the margins where pigments can adhere. Marginal staining depends on the efficacy of the adhesive/cementation system to bond the restoration to dental hard tissue(s) and individual patient factors [41]. The latter include nutritional habits such as consumption of coffee, black tea, or red wine as well as smoking and oral hygiene procedures [89, 90]. Additionally, the individual intraoral microbiome may play a role [91–93]. Less important is the restorative material [94–96] or the chosen operative technique [97, 98]. Nevertheless, there is evidence that suggests that the occurrence of marginal discolouration correlates with a compromised integrity of the marginal seal [47, 99], which may be frequently related to polymerization shrinkage of the composite.

### Colour match (category A3)

This category is applicable to tooth-colored restorations only. An ideal color match is achieved when all visually apparent differences between dental hard tissues and the restorative material are minimal or even invisible. Deviations in shade, translucency, or opacity between dental hard tissues and the restorative material are possible if (1) the chosen color of the restorative material does not match that of the surrounding dental hard tissues, (2) the natural teeth become darker or more yellow with increasing age [100], and (3) the restorative material itself has inherent color instability [101–104]. When color matching has to be evaluated, visual examination is the method of choice. In addition, intraoral photographs can be used but are also difficult to standardize during follow-up examinations [105, 106]. In contrast, commercially available color measuring instruments, e.g., reflectance spectrophotometers and colorimeters, have gained acceptance due to their satisfactory accuracy, reliability, and time-efficient use [86, 107–109].

## Domain M: miscellaneous

The expert panel decided to streamline the “core” FDI criteria set and additional methods are listed in Table 3. The patient’s view on the restored tooth as well as the radiographic assessment of restorations was shifted in a new domain. With respect to the impossibility to embed the corresponding diagnostic scores into the standard 5-point scale, both categories are shown in the illustrated version only, which can be downloaded from the journal’s website.

### Patient’s view (category M1)

Patient satisfaction with a dental restoration is a subjective response that gains more attention in practice-based or health service research and is usually scored by means of visual analogue scales [e.g., 113]. From the methodological point of view, it might be sufficient to ask for an overall (subjective) impression from the patient. In cases of dissatisfaction, a detailed report about pain, hypersensitivity, chewing comfort, occlusion, proximal contacts, cleanability, contours, or aesthetics might be of value. This assessment can be designed by use of the FDI criteria but a standardized protocol is not established or published so far. The patient’s opinion might be relevant, especially if the aesthetics of the restorations appear to be unacceptable for him/her and a replacement ahead of time needs to be discussed. It should be noted that the patient’s view can interfere with dental assessment and clinical decision-making.

### Assessment of dental restoration on radiographs (category M2)

In general, it has to be emphasized that there is no general justification to do a radiographic examination for the assessment of dental restorations without any clinical indication [111–114]. This approach is strictly in line with basic principles of radiation protection [115–117]. Nevertheless, the assessment of direct and indirection restorations is required on justified images. Here, radiographic evaluation includes, among others, caries detection, negative/positive steps or marginal gaps of the restoration, apical periodontitis, periodontal bone loss, internal/external resorption, or quality of endodontic treatment.

## Interpretation of the scorings

In addition to the intention to objectify the diagnostic evaluation and assessment of dental restorations and assist clinicians in decision-making, it is also important to consider clinically relevant key information, e.g., caries risk and activity, age, and medical or behavioral problems. On the basis of this comprehensive information, an individual intervention strategy has to be justified and agreed upon between the dentist and the patient/caregivers knowing well that the final decision might also be influenced by varying diagnoses, treatment philosophies, experiences, settings, and available resources including treatment costs. Importantly, each decision must be made with respect for the patient’s autonomy. Therefore, a specific dental diagnosis might be linked with different decisions.

With the revised FDI criteria set (Table 4), some ambiguities were removed, and scores were further harmonized to cross-link distinct clinical situations with possible management strategies, e.g., monitoring/reviewing (scores 1–4), refurbishment or reseal (score 3), repair (score 4), or replacement (score 5). An important issue is the decision whether a restoration is clinically acceptable (scores 1–3) or not (scores 4 and 5) and to decide further whether repair is possible (score 4) or not (score 5). Again, as described above, treatment procedures have to be understood as *possible* intervention corridors, and they are not meant to be understood as inevitable treatment approaches. In that respect it may be also good to consider contemporary tendencies in restorative dentistry to be as conservative/preservative as possible and, in case of doubt, rather select shorter monitoring intervals or the most minimally invasive option [118]. In this context, repair of direct and indirect restorations has to be considered a conservative treatment option in comparison to traditional replacement.

**Table 4:** Description and details of the revised FDI criteria set to evaluate direct and indirect dental restorations

	Functional properties (domain F)		
	F1: fracture of material and retention FDI 2010 category: 5	F2: marginal adaptation FDI 2010 category: 6	F3: proximal contact point FDI 2010 category: 8
Criteria	*Visual examination and short air drying*	*Visual examination, short air drying,* *and 250-µm probe*	*Visual examination and 25-/50-/100-µm blades*
1. Clinically excellent/very good (sufficient)	Restoration is completely present without deficiencies detectable after air drying. No crack, chipping/delamination, or material bulk fracture	Ideal marginal adaptation of the restoration at the dental hard tissue after air drying. No marginal gap detectable by gentle probing	Ideal contact point: 25-µm metal blade can pass through proximal contact and no inflammation of the gingiva/periodontium due to the proximal restoration. No food impaction
2. Clinically good (sufficient)	Restoration is completely present with minor deficiencies detectable after air drying, e.g., insignificant material chipping or one hairline crack	Slight deficiencies of marginal adaptation after air drying. Minor, superficial marginal gap(s) or ditching	Slightly weak contact point: 50-µm metal blade can pass through proximal contact and no inflammation of the gingiva/periodontium due to the proximal restoration. No food impaction
3. Clinically satisfactory (sufficient)	Restoration is present with deficiencies detectable without air drying, e.g., hairline cracks or distinct material loss (chipping). Material loss can mainly be corrected by refurbishment if needed	Distinct deficiencies of marginal adaptation without air drying: marginal gap(s) or ditching (width < 250 µm and/or depth < 2 mm)	Oversized contact point or excessive material: 25-µm metal blade cannot pass through proximal contact and inflammation of the gingiva/periodontium due to the proximal restoration. Refurbishment is possible. OR Severely weak contact point: 100-µm metal blade can pass through proximal contact but no inflammation of gingiva or discomfort
4. Clinically unsatisfactory (partially insufficient)	Localized but severe deficiencies regarding fracture and retention, e.g., chipping/delamination which cannot be refurbished, bulk fracture, or partially loose/lost restoration. Repair is possible. Lost indirect restoration, which can be recemented/reluted, is considered here	Localized but severe deficiencies of marginal adaptation: width ≥ 250 µm and/or depth ≥ 2 mm marginal gap(s). Partially loose/lost restoration. Repair is possible	Severely weak contact point: 100-µm metal blade can pass through proximal contact or unintended interlocked contact point. Inflammation of the gingiva/periodontium due to the proximal restoration and/or food impaction. Repair is possible
5. Clinically poor (entirely insufficient)	Generalized severe deficiencies, e.g., extensive delamination, multiple bulk fractures, or (nearly) completely loose/lost restoration. Repair not possible/reasonable	Generalized and severely compromised marginal adaptation: width ≥ 250 µm and/or depth ≥ 2 mm. Complete loose/lost restoration. Repair not possible/reasonable	Severely weak contact point: 100-µm metal blade can easily pass through proximal contact or unintended interlocked contact point (impossible to pass). Inflammation of the gingiva/periodontium due to the proximal restoration and/or food impaction. Repair not possible/reasonable
Not applicable	This code is used if examination for any reason is not possible		
Additional comments	1) Should be included without exception in any study that requires restoration assessment. 2) If a restoration is graded as entirely insufficient (F1/score 5) or completely lost all other functional (except F2) and aesthetical categories become not applicable	1) Evaluate gap formation at the restoration margin only. 2) If any loss of restoration material or dental hard tissue is evident, these findings have to be scored in the categories F1 and B2. Caries at the restoration margin has to be scored in category B1. 3) If a restoration is graded as entirely insufficient or completely lost (F2/score 5), all other functional and aesthetical categories become not applicable (except indirect restorations which can be recemented/reluted)	1) Not applicable in case of missing adjacent teeth, gap-toothed/flared/mobile dentition, or atypical individual tooth form, e.g., microdens or diastema. 2) Do not mix-up with F1

	Functional properties (domain F)	
	F4: form and contour FDI 2010 category: 4, 6, 7, 8	F5: occlusion and wear FDI 2010 category: 7
Criteria	*Visual examination*	*Visual examination and articulation paper*
1. Clinically excellent/very good (sufficient)	Outline, contour, convexity, embrasure, and/or marginal ridges are restored ideally in comparison to the individual, age-related and functional anatomy. No marginal step detectable by gentle probing	Ideal individual and age-related static and dynamic occlusion with multiple antagonistic contact points. No premature contacts, non-/hyper-occlusion, and/or balancing interferences
2. Clinically good (sufficient)	Minor deviations in outline, contour, convexity, embrasure, and/or marginal ridges in comparison to the individual, age-related and functional anatomy, AND/OR minor marginal steps, overhangs detectable by gentle probing	Minor deviations in individual and age-related static and dynamic occlusion with at least one antagonistic contact point per tooth. No premature contacts, non-/hyper-occlusion, and/or balancing interferences
3. Clinically satisfactory (sufficient)	Outline, contour, convexity, embrasure, and/or marginal ridges are distinctly misshaped but clinically acceptable and/or distinct negative/positive steps, overhangs. Refurbishment (removal of overhangs/steps) to some extent is possible	Hyper-occlusion, premature contacts, and/or balancing interferences that can be eliminated by refurbishment
4. Clinically unsatisfactory (partially insufficient)	Outline, contour, convexity, embrasure, and/or marginal ridges are in parts severely undersized in comparison to the individual, age-related, and functional anatomy AND/OR prominently negative marginal steps. Repair is possible	Localized, flat occlusal structure with severe non-occlusion AND/OR severely worn restoration. Repair is possible
5. Clinically poor (entirely insufficient)	Outline, contour, convexity, embrasure, and/or marginal ridges are generally and severely under- or oversized in comparison to the individual, age-related, and functional anatomy. Repair not possible/reasonable	Generalized, severe non-occlusion AND/OR extensively worn restoration. Repair not possible/reasonable
Not applicable	This code is used if examination for any reason is not possible	
Additional comments	1) Describes in particular the dentist’s or dental technician’s ability to restore the tooth in comparison to contralateral (unrestored) teeth. 2) Do not mix-up with F1, F2, or F3	1) Not applicable in case of irregular individual tooth form or malocclusion, e.g., microdens or missing antagonistic teeth. 2) In case of severe and generalized fracture and retention deficiencies of a restoration (F1/score 5), score 5 (F5) is becoming not applicable. 3) Do not mix-up with F1

	Biological properties (domain B)		
	B1: caries at restoration margin (CAR) FDI 2010 category: 12	B2: dental hard tissue defects at restoration margin FDI 2010 category: 13	B3: postoperative hypersensitivity/pulp status FDI 2010 category: 11
Criteria	*Visual examination, short air drying,* *and 250-µm probe*	*Visual examination*	*Tooth hypersensitivity reported by patient; pulp sensitivity tested with cold stimulus*
1. Clinically excellent/very good (sufficient)	No caries/demineralization at the restoration margin detectable after air drying	Intact dental hard tissue without crack lines and fractures at the restoration margin	No postoperative hypersensitivity or pain on chewing and/or cold/warm food items reported by the patient. Normal (short) reaction to sensitivity test on cold
2. Clinically good (sufficient)	First visible signs of a non-cavitated caries lesion at the restoration margin detectable after air drying	Minor vertical/horizontal hairline crack lines in enamel at the restoration margin	Patient reports minor postoperative hypersensitivity or minor pain on chewing and/or cold/warm food items reported by the patient for a limited period of time (< 1 week). Normal (short) reaction to sensitivity test on cold
3. Clinically satisfactory (sufficient)	Established, non-cavitated caries lesion or microcavity at the restoration margin detectable without air drying	Distinct enamel chipping or enamel fracture at the restoration margin. If necessary, deficiencies can be corrected by refurbishment	Patient reports distinct postoperative hypersensitivity or distinct pain on chewing and/or cold/warm food items reported by the patient for a prolonged period of time (> 1 week). Normal (short) or more intense reaction to sensitivity test on cold
4. Clinically unsatisfactory (partially insufficient)	Localized dentin cavity (width > 250 µm, depth > 2 mm) at the restoration margin. Repair is possible	Severe marginal (enamel) fracture, partially fractured cusp or ridge at the restoration margin. Repair is possible	Patient reports severe/persistent, postoperative hypersensitivity or persistent pain on chewing and/or cold/warm food items reported by the patient for a prolonged period of time (> 1 month) AND/OR intense reaction to sensitivity test on cold. Both symptoms indicate irreversible pulpitis. Endodontic treatment requires access cavity only
5. Clinically poor (entirely insufficient)	Extensive dentin cavity at the restoration margin. Repair not possible/reasonable	Cusp or tooth fracture, e.g., involving enamel, dentin, and cementum possible with mobile fragments/pain when biting OR cracked tooth syndrome related to restoration. Repair not possible/reasonable	Irreversible pulpitis, nonvital tooth, pulp necrosis with or without periapical periodontitis after restoration placement. Endodontic treatment requires replacement of the restoration
Not applicable	This code is used if examination for any reason is not possible		
Additional comments	1) Do not confuse caries with marginal staining (A2). 2) Consider only caries lesions that are located directly at the restoration margin. 3) If any loss of restoration material or dental hard tissue is evident, these findings have to be scored in the corresponding categories F1 and B2	1) Do not misdiagnose attrition, erosive tooth wear, etc. in this category. 2) If loss of restoration material or CAR is evident, these entities have to be scored in the corresponding categories F1 and B1	1) This category can only be evaluated in vital teeth that are monitored from the time the restoration is placed. 2) Refurbishment, repair, or replacement cannot be related to a possible endodontic treatment procedure; therefore, possible restorative interventions are not used for categorization

	Aesthetic properties (domain A)		
	A1: surface luster and surface texture FDI 2010 category: A1	A2: marginal staining FDI 2010 category: A2	A3: color match FDI 2010 category: A3
Criteria	*Visual examination and short air drying*	*Visual examination and short air drying*	*Visual examination*
1. Clinically excellent/very good (sufficient)	Surface luster and surface texture comparable to dental hard tissue/adjacent teeth after air drying	No marginal staining detectable after air drying	No deviation in shade, translucency/opacity between restoration, and neighboring dental hard tissue/adjacent teeth
2.Clinically good (sufficient)	Slightly dull surface luster and/or surface texture with minor deviations, e.g., isolated/small marks, pores, and/or voids detectable compared to dental hard tissue/adjacent teeth after air drying	Minor marginal staining detectable after air drying	Minor deviation in shade, translucency/opacity between restoration, and neighboring dental hard tissue/adjacent teeth detectable
3. Clinically satisfactory/acceptable (sufficient)	Dull surface luster and/or surface texture with distinct deviations, e.g., marks, pores, and/or voids detectable compared to dental hard tissue/adjacent teeth detectable without air drying. Refurbishment is possible	Distinct marginal staining detectable without air drying but not displeasing. Refurbishment is possible	Distinct deviation in shade, translucency/opacity between restoration, and neighboring dental hard tissue/adjacent teeth detectable but not displeasing
4. Clinically unsatisfactory (partially insufficient)	Localized, displeasing dull surface luster and/or rough surface texture with substantial deviations/multiple pores/voids detectable compared to dental hard tissue/adjacent teeth which can be repaired	Localized, displeasing deep marginal staining. Marginal staining can be removed/improved by repair	Localized, displeasing deviation in shade, translucency/opacity between restoration, and neighboring dental hard tissue/adjacent teeth which can be improved by repair
5. Clinically poor (entirely insufficient)	Generalized, displeasing dull surface luster and/or rough surface texture with substantial deviations/multiple pores/voids compared to dental hard tissue/adjacent teeth. Repair not possible/reasonable	Generalized, displeasing deep marginal staining. Repair not possible/reasonable	Generalized, displeasing deviation in shade, translucency/opacity between restoration, and neighboring dental hard tissue/adjacent teeth. Repair not possible/reasonable
Not applicable	This code is used if the restoration is mostly or fully lost or loose and/or examination for any other reason is not possible		
Additional comments	1) The evaluation of aesthetic properties is relevant for decision making on tooth-coloured restorations in visible tooth surfaces only. 2) Evaluation can be performed from a standard examination distance under operating light (~ 40 cm) or from speaking distance (~ 80–100 cm) with the operating light switched off. This has to be defined and reported later		
	If surface luster and surface texture have to be taken in account, the worse characteristic determines the grading	Do not confuse marginal staining with CAR (B1)	Evaluation of tooth-colored restorations only

## Reporting of future studies

In addition to the detailed description of the FDI criteria set and the clinical interpretation of the diagnostic findings, it is vital to highlight the need for standardized study reporting that includes the evaluation of dental restorations. A reporting checklist is given in Table 5, which should help researchers to standardize their paper writing.

**Table 5:** Reporting checklist for studies evaluating direct and indirect restorations

Section and topic	Item no	Recommendations
Title	1	Indicate the type of study and important parameters (material, Black’s classification, primary or permanent teeth)
Abstract	2	Structured summary of study design, methods, results, and conclusions
Introduction		Introduce and justify
Background/rationale	3	Scientific and clinical background including the intended indication and clinical use of the restorative procedures and/or materials. Justify research needs
Objectives	4	Study objectives and hypotheses
Methods		Describe
Study design	5	Indicate the study design, e.g., randomized controlled clinical trial, clinical trial, case control study, case study, or epidemiological, observational, or diagnostic study, and if the study protocol was designed prospectively or retrospectively. Describe the study setting, e.g., university- or practice-based and the allocation of interventions, e.g., split-mouth or parallel-arm design. Report relevant dates, time intervals including periods of recruitment, and follow-ups
Sample size	6	Intended sample size and how it was determined (sample size calculation). Include typically no more than one restoration per study arm
Patients	7	Patient population, e.g., children, adolescents, adults, elderly, etc. In- and exclusion criteria for patients, procedures for screening, selection, and randomization. Where and when potentially eligible participants were identified (setting, location, and dates). Give the eligibility criteria, screening, and selection procedures. Report numbers of individuals in each stage of study, e.g., numbers potentially eligible, examined for eligibility, confirmed eligible, included in the study, completing follow-up, and analyzed. Give reasons for nonparticipation or drop-out in each stage
Teeth	8	Exclusion and inclusion criteria for tooth selection, cleaning, and processing prior to beginning of the study/placing restoration. Account for all teeth that were included, restored, and monitored. Report the number of included teeth separately for each type of restoration, e.g., according to Black’s classification, etc. Report indications or reasons for restoration, tooth type, number of restored surfaces, and tooth-related dental history, i.e., caries, endodontic treatment, or trauma
Restorative interventions and materials	9	Provide complete and detailed information about the clinical setting, workflow, instruments, and materials (product and batch number, manufacturer) for all restorative procedures. Indicate operator initials
Evaluation of restorations	10a	Provide complete and detailed information about the evaluation setting, workflow, illumination, cleaning, drying, instruments, and procedures for repeated diagnostic evaluation of all restorations. Indicate examiner initials
	10b	Define primary and secondary outcome parameters. Report and describe which categories of the FDI criteria set were selected and why and how they were used
	10c	Report and describe if additional clinical or laboratory evaluations were performed by which researcher, e.g., sensibility testing, intraoral photographs, 3D wear analysis on digital models, or scanning electron microscopy analysis of marginal adaptation on replica models, etc
Blinding	11	Indicate if operator(s), examiner(s), patients, and statistician(s) were blinded or if an independent evaluation procedure was included, e.g., on photographs
Training and calibration	12	Details of theoretical and practical training, training setting, and results from calibration for operators and examiners, e.g., Kappa values, should be given
Operators and examiners	13	Report the role and level of clinical and/or diagnostic (research) experience of each operator and examiner, e.g., years of relevant clinical experience. Visual acuity of both operators and examiners should be reported
Data handling and statistics	14	Describe all statistical methods for evaluating the longevity of restorations and its quality over time including descriptive data for each of the chosen categories. Explain how variables and missing data were handled in the analyses. Indicate the used statistical methods to analyze the survival probability, e.g., Kaplan–Meier statistics/curves, and to compare different groups, e.g., log-rank test, Wilcoxon signed rank test, Bonferroni corrections, multivariate analyses or Cox regression, or proportional hazards models
Results		Report
Study population and/or teeth	15	Flow of participants, using a diagram. Report numbers of the included patients and teeth in relation to test and control groups
Characterization of the study population	16	Characterize the study population (age, female/male ratio, dental health status, oral hygiene, etc.)
Outcome data	17a	Report adverse events and undesirable effects
	17b	Provide complete descriptive and explorative data of quality and longevity of tested restorations. Kaplan–Meier statistics/curves illustrate the cumulative survival probability over the study period
	17c	Present results from comparative analysis
Discussion		Discuss
Study population	18	Conclude whether the study population is representative for the target group. Furthermore, include a statement if the study sample met the requirements from the sample size calculation. Evaluate dropout and attrition rates
Data interpretation	19	Summarize the important findings from the study and interpret the data in relation to the recently published literature. Consider potential methodological differences between studies and its influence on the comparability. Furthermore, discuss the (clinical) relevance of the study results and the potential implications for dental practice. Compare the results with those of similar clinical studies and assess deviations if present
Strength and limitation	20	Consider methodological strengths and limitations of the used study design. Report potential sources of bias, statistical uncertainty, and lacking generalizability. Discuss both direction and magnitude of any potential bias
Conclusion	21	Draw a well-balanced and unbiased study conclusion
Other information		If applicable
Ethics	22	Indicate the ethical committee/institutional review board and trial registration number
Funding	23	Mention sources of funding and other support. Explain the role of funders
Conflict of interest	24	Summarize potential conflicts of interest for each of the authors

## Conclusions

The formerly published FDI criteria set for the evaluation of direct and indirect restorations [1–5] was revisited through a stepwise consensus process. With the aim of improving clinical usability, practicability, and acceptability, a revised set of criteria prioritized categories and harmonized the wording. It is also important that each domain or category can be selected independently, thus creating a modular diagnostic system with great flexibility for the evaluation of direct and indirect restorations. The revised FDI criteria set has to be understood as a living document that can be regularly adopted on the basis of new clinical data, findings and experiences. Therefore, we encourage researchers, teachers, and dental practitioners to provide feedback.

## Supplementary Information

Below is the link to the electronic supplementary material.

Supplementary file1 An illustrated version of the FDI criteria set can be downloaded from the journal website. (PPTX 24.5 MB)
