# Correction to: Revised FDI criteria for evaluating direct and indirect dental restorations—recommendations for its clinical use, interpretation, and reporting

**DOI:** 10.1007/s00784-022-04851-w

**Published:** 2023-01-06

**Authors:** Reinhard Hickel, Sabine Mesinger, Niek Opdam, Bas Loomans, Roland Frankenberger, Milena Cadenaro, John Burgess, Arnd Peschke, Siegward D. Heintze, Jan Kühnisch

**Affiliations:** 1 Department of Conservative Dentistry and Periodontology, University Hospital, Ludwig-Maximilians-University of Munich, Munich, Germany; 2 Department of Dentistry, Radboud Institute for Health Sciences, Radboud University Medical Center, Nijmegen, The Netherlands; 3 Department of Operative Dentistry, Endodontics, and Pediatric Dentistry Medical Center for Dentistry, University Medical Center Giessen and Marburg, Campus Marburg, Marburg, Germany; 4 Department of Medical Sciences, University of Trieste, Trieste, Italy and Children’s Hospital “Burlo Garofolo,” Institute for Maternal and Child Health, Trieste, Italy; 5 Louisiana State University Health Sciences Center, New Orleans, LA USA; 6 IvoclarVivadent AG, Research & Development, Schaan, Liechtenstein

**Correction to: Clinical Oral Investigations**

10.1007/s00784-022-04814-1

In the published paper, the supplementary file is containing an additional page, which was not intended to make public. Unfortunately, it was made online during the publication process.

The original article has been corrected.

