# Supplementary material for: Revised FDI criteria for evaluating direct and indirect dental restorations—recommendations for its clinical use, interpretation, and reporting
Source: Clin Oral Investig. 2022 Dec 12;27(6):2573–92. doi: 10.1007/s00784-022-04814-1 (PMC10264483; doi:10.1007/s00784-022-04814-1)
Supplement: Supplementary file 1 — Supplementary file1 An illustrated version of the FDI criteria set can be downloaded from the journal website. (PPTX 24.5 MB) [file 784_2022_4814_MOESM1_ESM.pptx]

## Slide 1
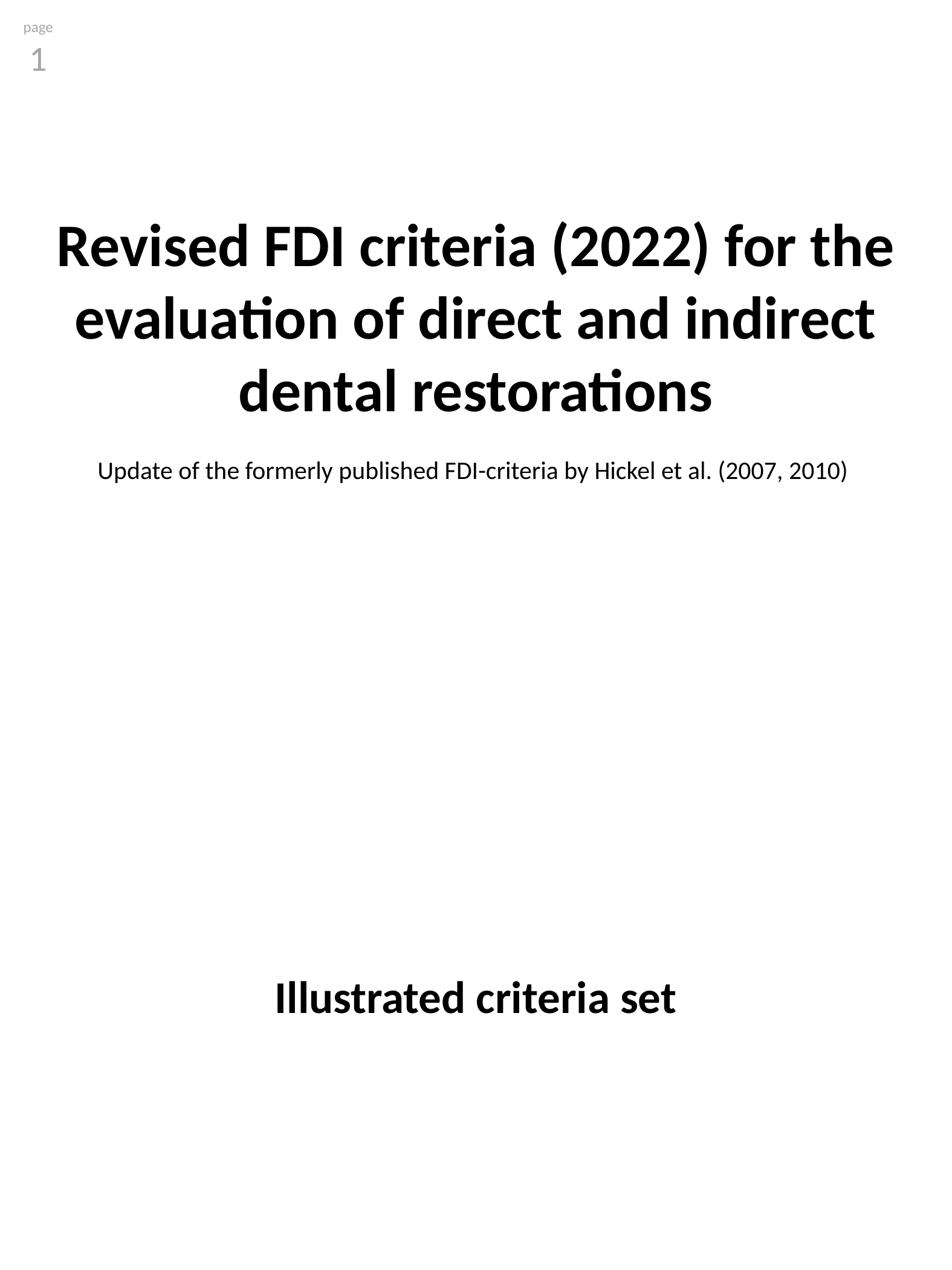

page
1
Revised FDI criteria (2022) for the evaluation of direct and indirect dental restorations
Update of the formerly published FDI-criteria by Hickel et al. (2007, 2010)
Illustrated criteria set

## Slide 2
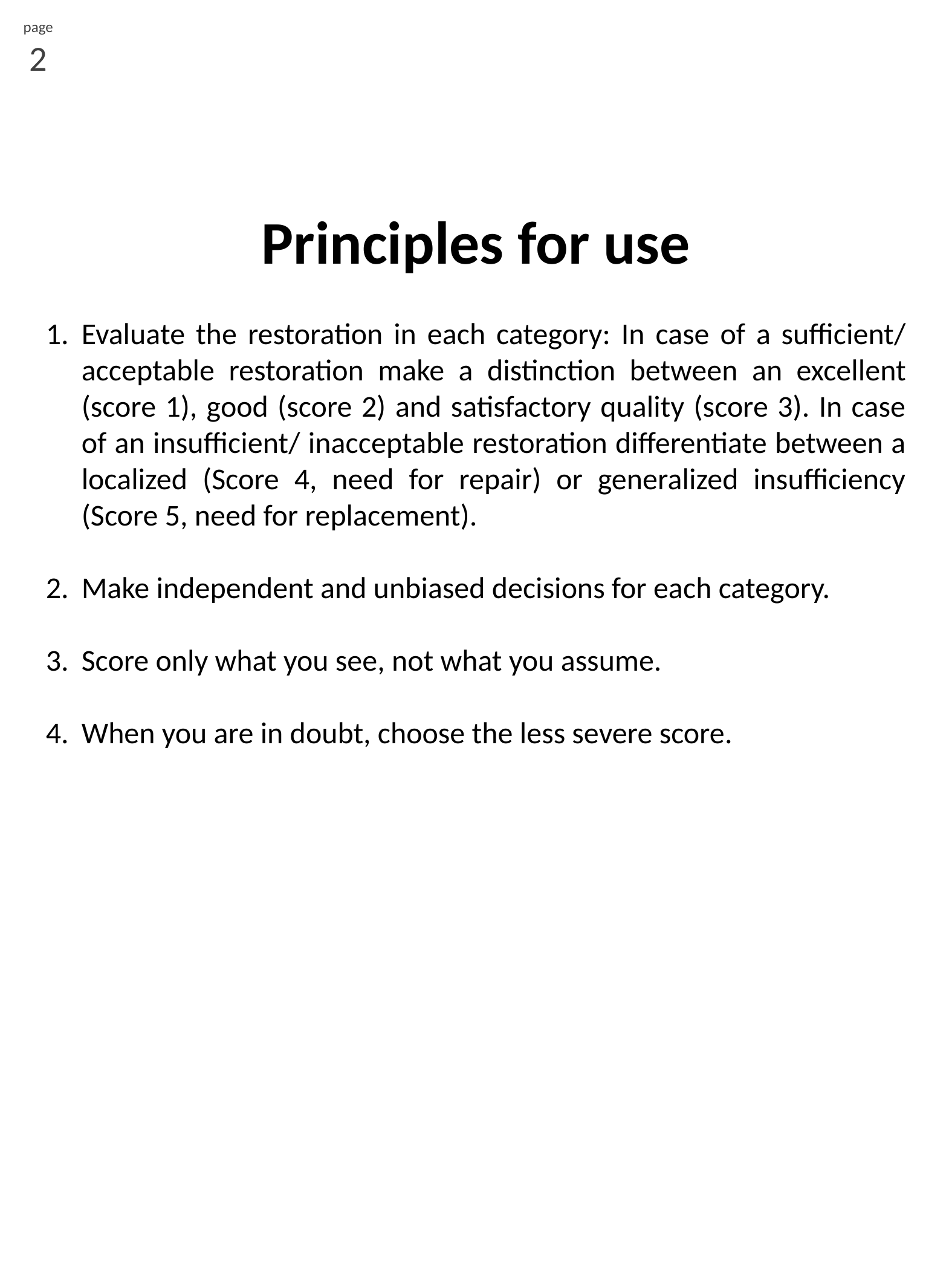

page
2
Principles for use
Evaluate the restoration in each category: In case of a sufficient/ acceptable restoration make a distinction between an excellent (score 1), good (score 2) and satisfactory quality (score 3). In case of an insufficient/ inacceptable restoration differentiate between a localized (Score 4, need for repair) or generalized insufficiency (Score 5, need for replacement).
2.	Make independent and unbiased decisions for each category.
3. 	Score only what you see, not what you assume.
4. 	When you are in doubt, choose the less severe score.

## Slide 3
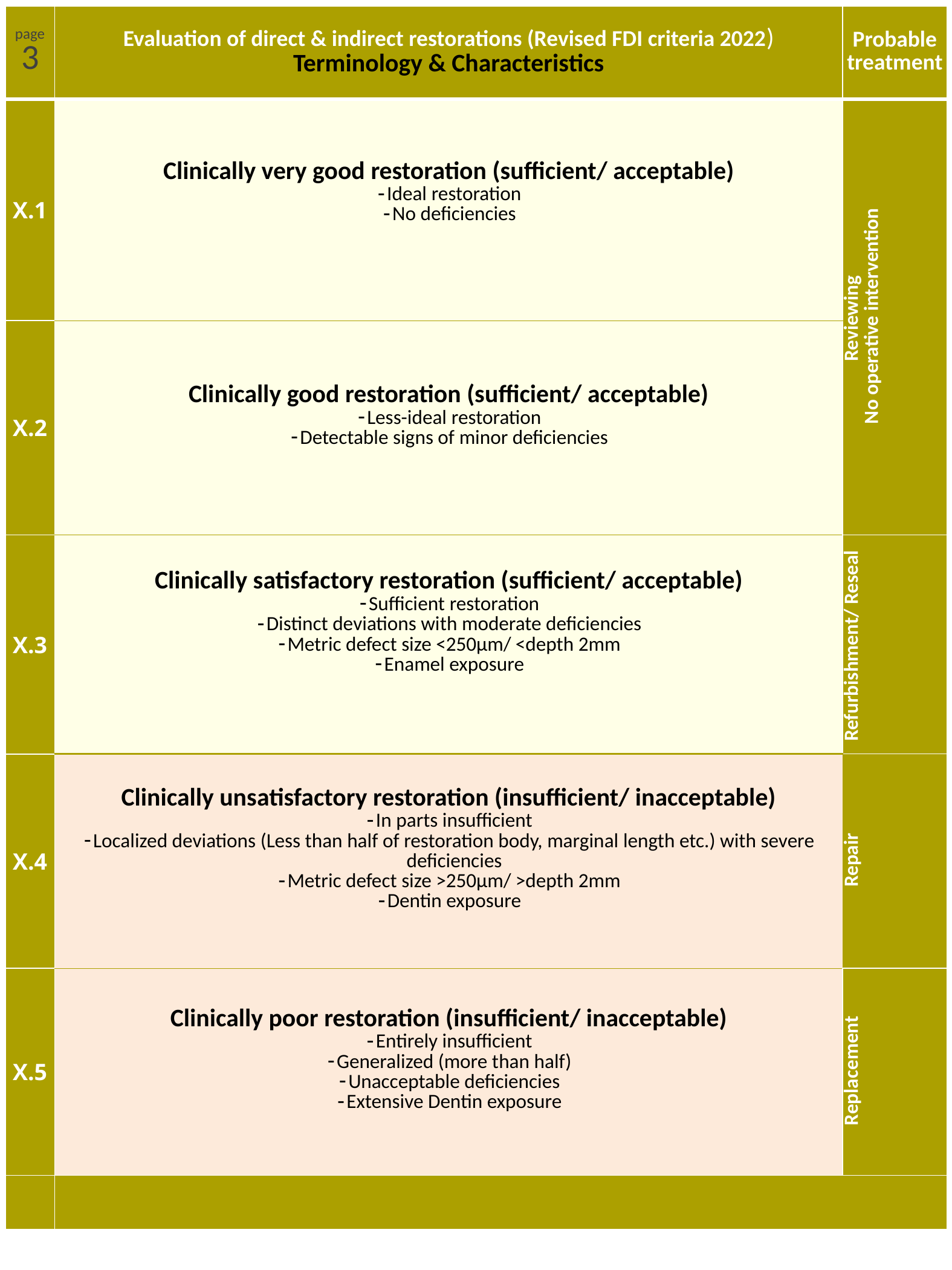

| page 3 | Evaluation of direct & indirect restorations (Revised FDI criteria 2022) Terminology & Characteristics | Probable treatment |
| --- | --- | --- |
| X.1 | Clinically very good restoration (sufficient/ acceptable) Ideal restoration No deficiencies | Reviewing No operative intervention |
| X.2 | Clinically good restoration (sufficient/ acceptable) Less-ideal restoration Detectable signs of minor deficiencies | |
| X.3 | Clinically satisfactory restoration (sufficient/ acceptable) Sufficient restoration Distinct deviations with moderate deficiencies Metric defect size <250µm/ <depth 2mm Enamel exposure | |
| | | Refurbishment/ Reseal |
| X.4 | Clinically unsatisfactory restoration (insufficient/ inacceptable) In parts insufficient Localized deviations (Less than half of restoration body, marginal length etc.) with severe deficiencies Metric defect size >250µm/ >depth 2mm Dentin exposure | Repair |
| X.5 | Clinically poor restoration (insufficient/ inacceptable) Entirely insufficient Generalized (more than half) Unacceptable deficiencies Extensive Dentin exposure | Replacement |
| | | |

## Slide 4
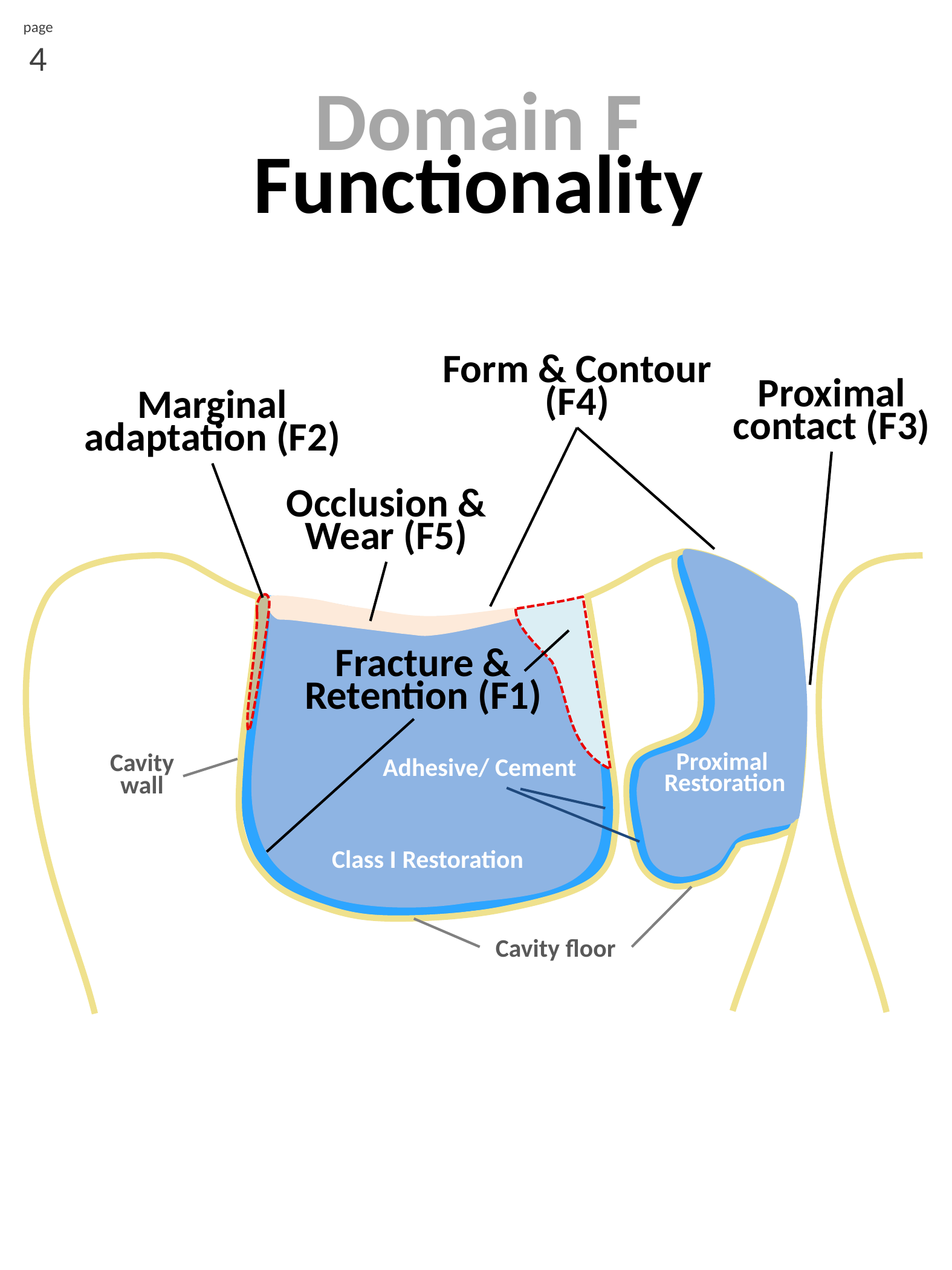

page
4
Domain F
Functionality
Form & Contour (F4)
Proximal contact (F3)
Marginal adaptation (F2)
Occlusion & Wear (F5)
Fracture & Retention (F1)
Adhesive/ Cement
Cavity wall
Proximal
Restoration
Class I Restoration
Cavity floor

## Slide 5
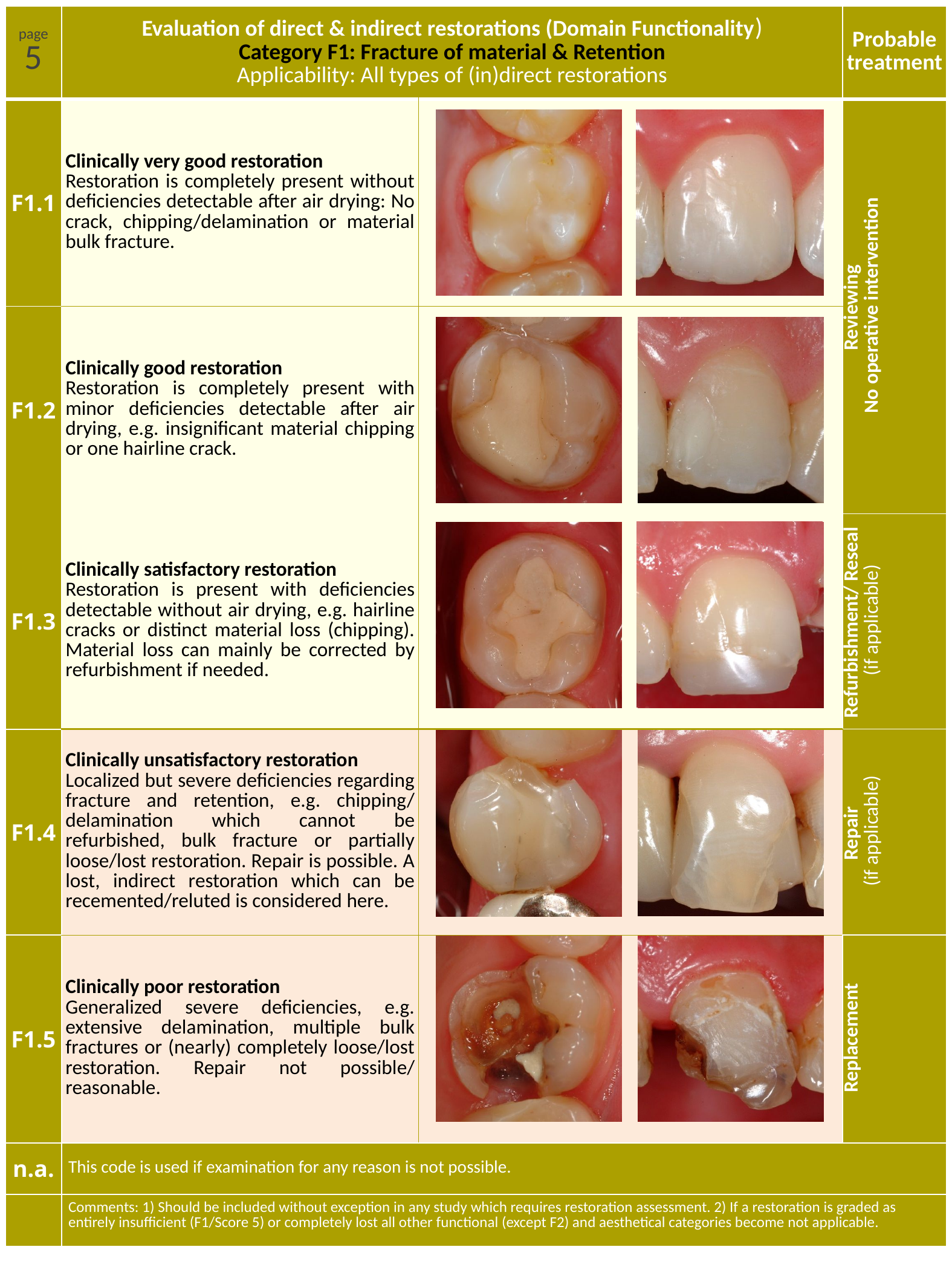

| page 5 | Evaluation of direct & indirect restorations (Domain Functionality) Category F1: Fracture of material & Retention Applicability: All types of (in)direct restorations | | Probable treatment |
| --- | --- | --- | --- |
| F1.1 | Clinically very good restoration Restoration is completely present without deficiencies detectable after air drying: No crack, chipping/delamination or material bulk fracture. | | Reviewing No operative intervention |
| F1.2 | Clinically good restoration Restoration is completely present with minor deficiencies detectable after air drying, e.g. insignificant material chipping or one hairline crack. | | |
| F1.3 | Clinically satisfactory restoration Restoration is present with deficiencies detectable without air drying, e.g. hairline cracks or distinct material loss (chipping). Material loss can mainly be corrected by refurbishment if needed. | | |
| | | | Refurbishment/ Reseal (if applicable) |
| F1.4 | Clinically unsatisfactory restoration Localized but severe deficiencies regarding fracture and retention, e.g. chipping/ delamination which cannot be refurbished, bulk fracture or partially loose/lost restoration. Repair is possible. A lost, indirect restoration which can be recemented/reluted is considered here. | | Repair (if applicable) |
| F1.5 | Clinically poor restoration Generalized severe deficiencies, e.g. extensive delamination, multiple bulk fractures or (nearly) completely loose/lost restoration. Repair not possible/ reasonable. | | Replacement |
| n.a. | This code is used if examination for any reason is not possible. | | |
| | Comments: 1) Should be included without exception in any study which requires restoration assessment. 2) If a restoration is graded as entirely insufficient (F1/Score 5) or completely lost all other functional (except F2) and aesthetical categories become not applicable. | | |

## Slide 6
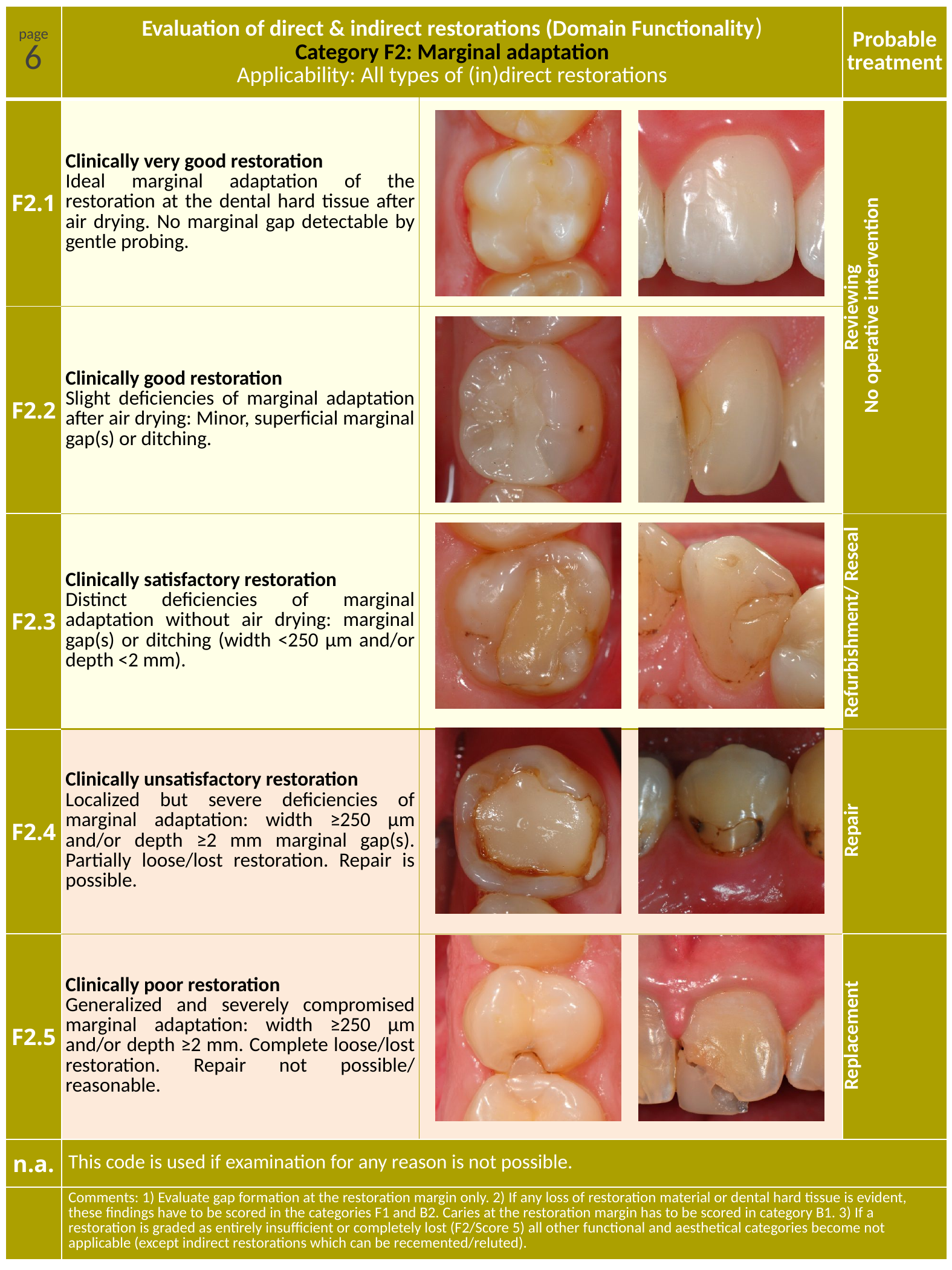

| page 6 | Evaluation of direct & indirect restorations (Domain Functionality) Category F2: Marginal adaptation Applicability: All types of (in)direct restorations | | Probable treatment |
| --- | --- | --- | --- |
| F2.1 | Clinically very good restoration Ideal marginal adaptation of the restoration at the dental hard tissue after air drying. No marginal gap detectable by gentle probing. | | Reviewing No operative intervention |
| F2.2 | Clinically good restoration Slight deficiencies of marginal adaptation after air drying: Minor, superficial marginal gap(s) or ditching. | | |
| F2.3 | Clinically satisfactory restoration Distinct deficiencies of marginal adaptation without air drying: marginal gap(s) or ditching (width <250 µm and/or depth <2 mm). | | Refurbishment/ Reseal |
| F2.4 | Clinically unsatisfactory restoration Localized but severe deficiencies of marginal adaptation: width ≥250 µm and/or depth ≥2 mm marginal gap(s). Partially loose/lost restoration. Repair is possible. | | Repair |
| F2.5 | Clinically poor restoration Generalized and severely compromised marginal adaptation: width ≥250 µm and/or depth ≥2 mm. Complete loose/lost restoration. Repair not possible/ reasonable. | | Replacement |
| n.a. | This code is used if examination for any reason is not possible. | | |
| | Comments: 1) Evaluate gap formation at the restoration margin only. 2) If any loss of restoration material or dental hard tissue is evident, these findings have to be scored in the categories F1 and B2. Caries at the restoration margin has to be scored in category B1. 3) If a restoration is graded as entirely insufficient or completely lost (F2/Score 5) all other functional and aesthetical categories become not applicable (except indirect restorations which can be recemented/reluted). | | |

## Slide 7
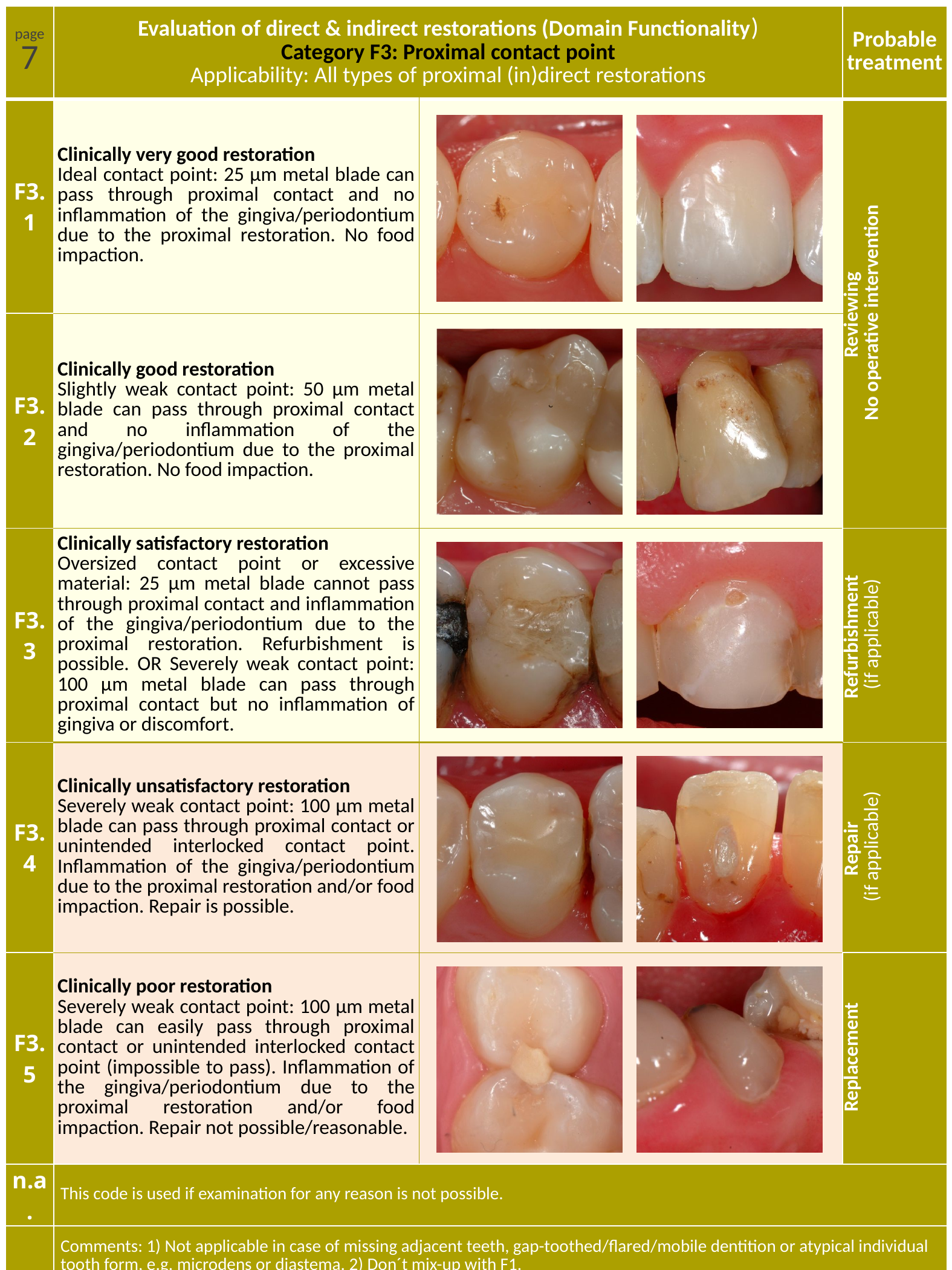

| page 7 | Evaluation of direct & indirect restorations (Domain Functionality) Category F3: Proximal contact point Applicability: All types of proximal (in)direct restorations | | Probable treatment |
| --- | --- | --- | --- |
| F3.1 | Clinically very good restoration Ideal contact point: 25 µm metal blade can pass through proximal contact and no inflammation of the gingiva/periodontium due to the proximal restoration. No food impaction. | | Reviewing No operative intervention |
| F3.2 | Clinically good restoration Slightly weak contact point: 50 µm metal blade can pass through proximal contact and no inflammation of the gingiva/periodontium due to the proximal restoration. No food impaction. | | |
| F3.3 | Clinically satisfactory restoration Oversized contact point or excessive material: 25 µm metal blade cannot pass through proximal contact and inflammation of the gingiva/periodontium due to the proximal restoration. Refurbishment is possible. OR Severely weak contact point: 100 µm metal blade can pass through proximal contact but no inflammation of gingiva or discomfort. | | Refurbishment (if applicable) |
| F3.4 | Clinically unsatisfactory restoration Severely weak contact point: 100 µm metal blade can pass through proximal contact or unintended interlocked contact point. Inflammation of the gingiva/periodontium due to the proximal restoration and/or food impaction. Repair is possible. | | Repair (if applicable) |
| F3.5 | Clinically poor restoration Severely weak contact point: 100 µm metal blade can easily pass through proximal contact or unintended interlocked contact point (impossible to pass). Inflammation of the gingiva/periodontium due to the proximal restoration and/or food impaction. Repair not possible/reasonable. | | Replacement |
| n.a. | This code is used if examination for any reason is not possible. | | |
| | Comments: 1) Not applicable in case of missing adjacent teeth, gap-toothed/flared/mobile dentition or atypical individual tooth form, e.g. microdens or diastema. 2) Don´t mix-up with F1. | | |

## Slide 8
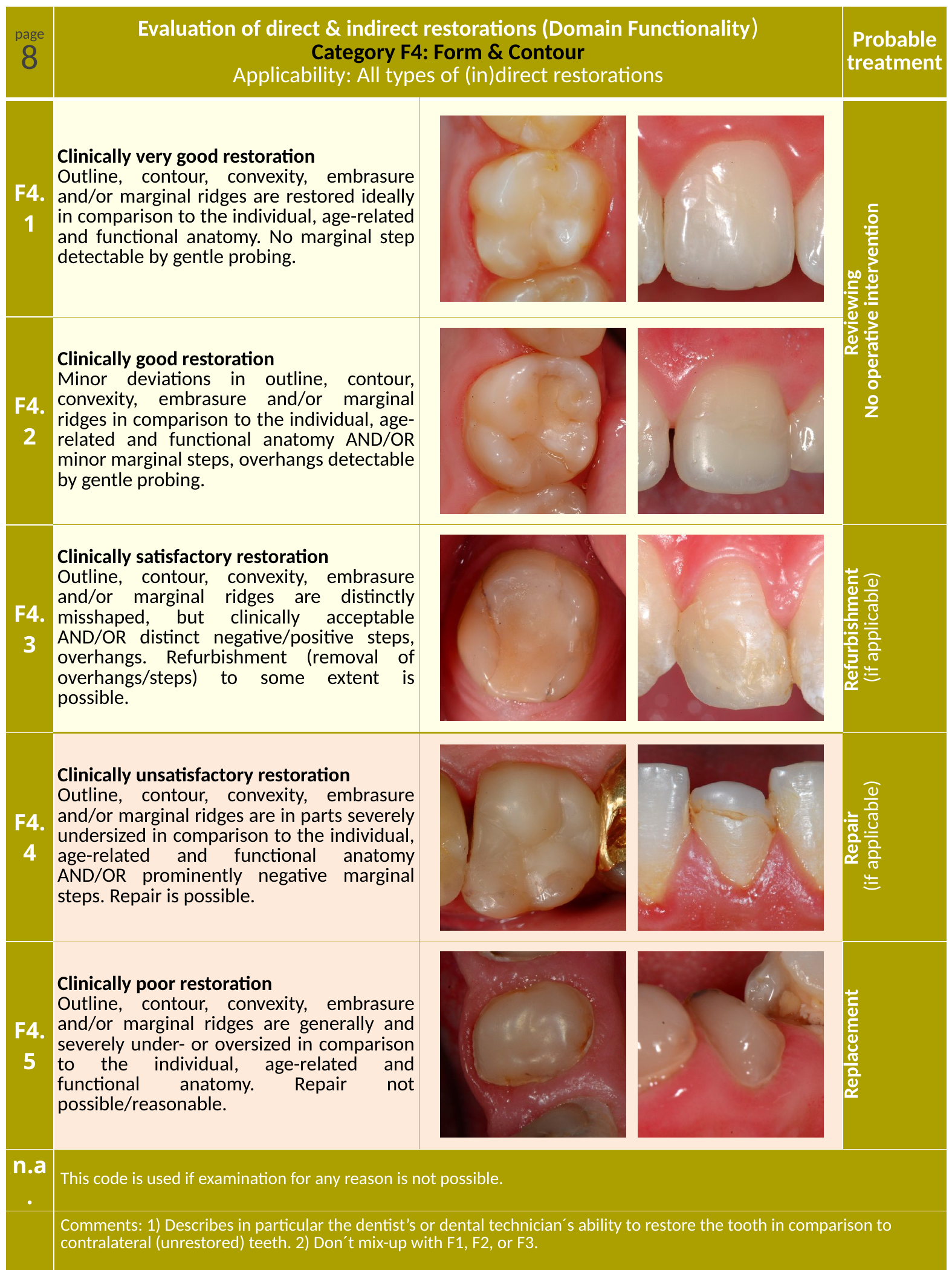

| page 8 | Evaluation of direct & indirect restorations (Domain Functionality) Category F4: Form & Contour Applicability: All types of (in)direct restorations | | Probable treatment |
| --- | --- | --- | --- |
| F4.1 | Clinically very good restoration Outline, contour, convexity, embrasure and/or marginal ridges are restored ideally in comparison to the individual, age-related and functional anatomy. No marginal step detectable by gentle probing. | | Reviewing No operative intervention |
| F4.2 | Clinically good restoration Minor deviations in outline, contour, convexity, embrasure and/or marginal ridges in comparison to the individual, age-related and functional anatomy AND/OR minor marginal steps, overhangs detectable by gentle probing. | | |
| F4.3 | Clinically satisfactory restoration Outline, contour, convexity, embrasure and/or marginal ridges are distinctly misshaped, but clinically acceptable AND/OR distinct negative/positive steps, overhangs. Refurbishment (removal of overhangs/steps) to some extent is possible. | | Refurbishment (if applicable) |
| F4.4 | Clinically unsatisfactory restoration Outline, contour, convexity, embrasure and/or marginal ridges are in parts severely undersized in comparison to the individual, age-related and functional anatomy AND/OR prominently negative marginal steps. Repair is possible. | | Repair (if applicable) |
| F4.5 | Clinically poor restoration Outline, contour, convexity, embrasure and/or marginal ridges are generally and severely under- or oversized in comparison to the individual, age-related and functional anatomy. Repair not possible/reasonable. | | Replacement |
| n.a. | This code is used if examination for any reason is not possible. | | |
| | Comments: 1) Describes in particular the dentist’s or dental technician´s ability to restore the tooth in comparison to contralateral (unrestored) teeth. 2) Don´t mix-up with F1, F2, or F3. | | |

## Slide 9
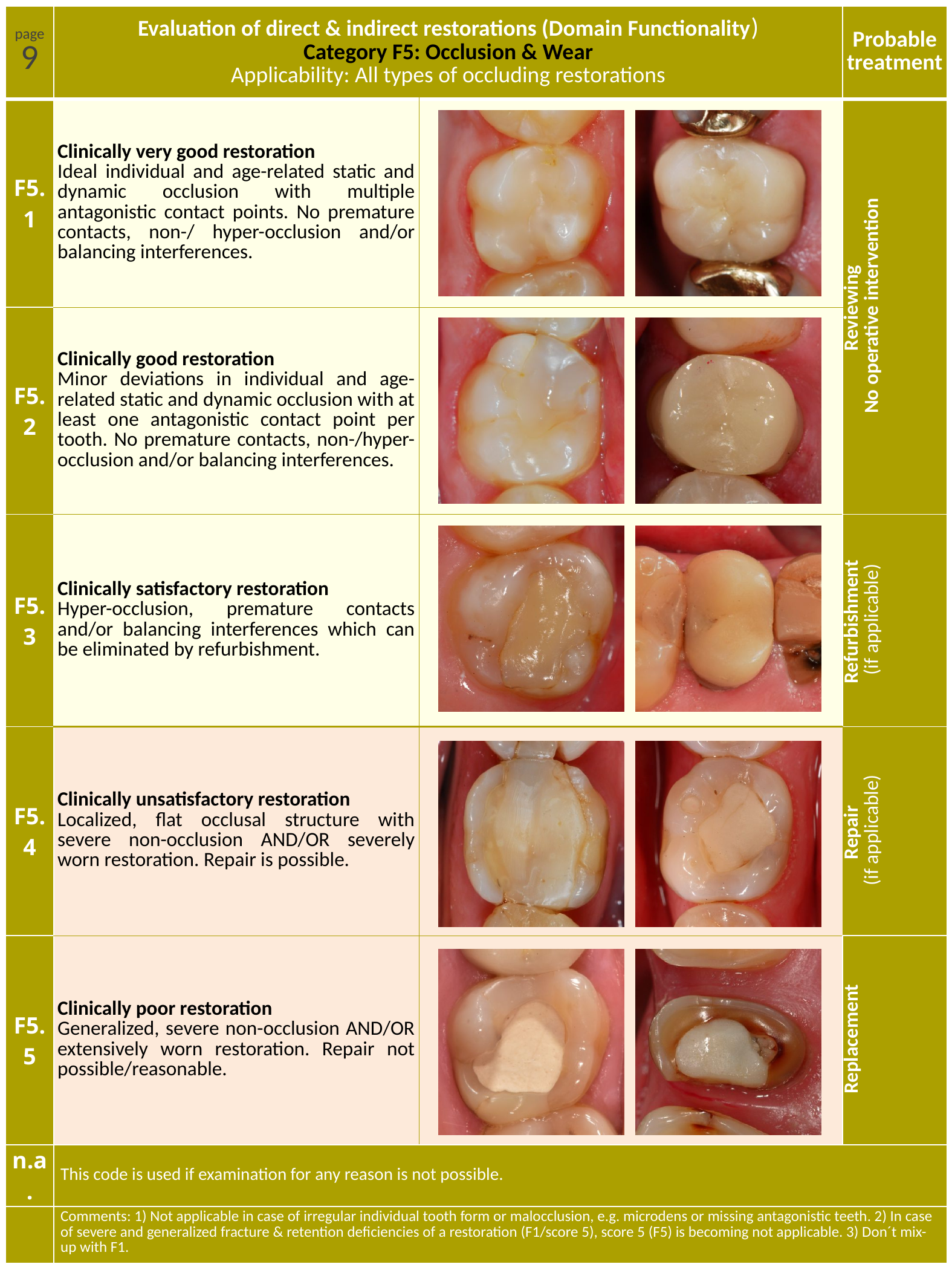

| page9 | Evaluation of direct & indirect restorations (Domain Functionality) Category F5: Occlusion & Wear Applicability: All types of occluding restorations | | Probable treatment |
| --- | --- | --- | --- |
| F5.1 | Clinically very good restoration Ideal individual and age-related static and dynamic occlusion with multiple antagonistic contact points. No premature contacts, non-/ hyper-occlusion and/or balancing interferences. | | Reviewing No operative intervention |
| F5.2 | Clinically good restoration Minor deviations in individual and age-related static and dynamic occlusion with at least one antagonistic contact point per tooth. No premature contacts, non-/hyper-occlusion and/or balancing interferences. | | |
| F5.3 | Clinically satisfactory restoration Hyper-occlusion, premature contacts and/or balancing interferences which can be eliminated by refurbishment. | | Refurbishment (if applicable) |
| F5.4 | Clinically unsatisfactory restoration Localized, flat occlusal structure with severe non-occlusion AND/OR severely worn restoration. Repair is possible. | | Repair (if applicable) |
| F5.5 | Clinically poor restoration Generalized, severe non-occlusion AND/OR extensively worn restoration. Repair not possible/reasonable. | | Replacement |
| n.a. | This code is used if examination for any reason is not possible. | | |
| | Comments: 1) Not applicable in case of irregular individual tooth form or malocclusion, e.g. microdens or missing antagonistic teeth. 2) In case of severe and generalized fracture & retention deficiencies of a restoration (F1/score 5), score 5 (F5) is becoming not applicable. 3) Don´t mix-up with F1. | | |

## Slide 10
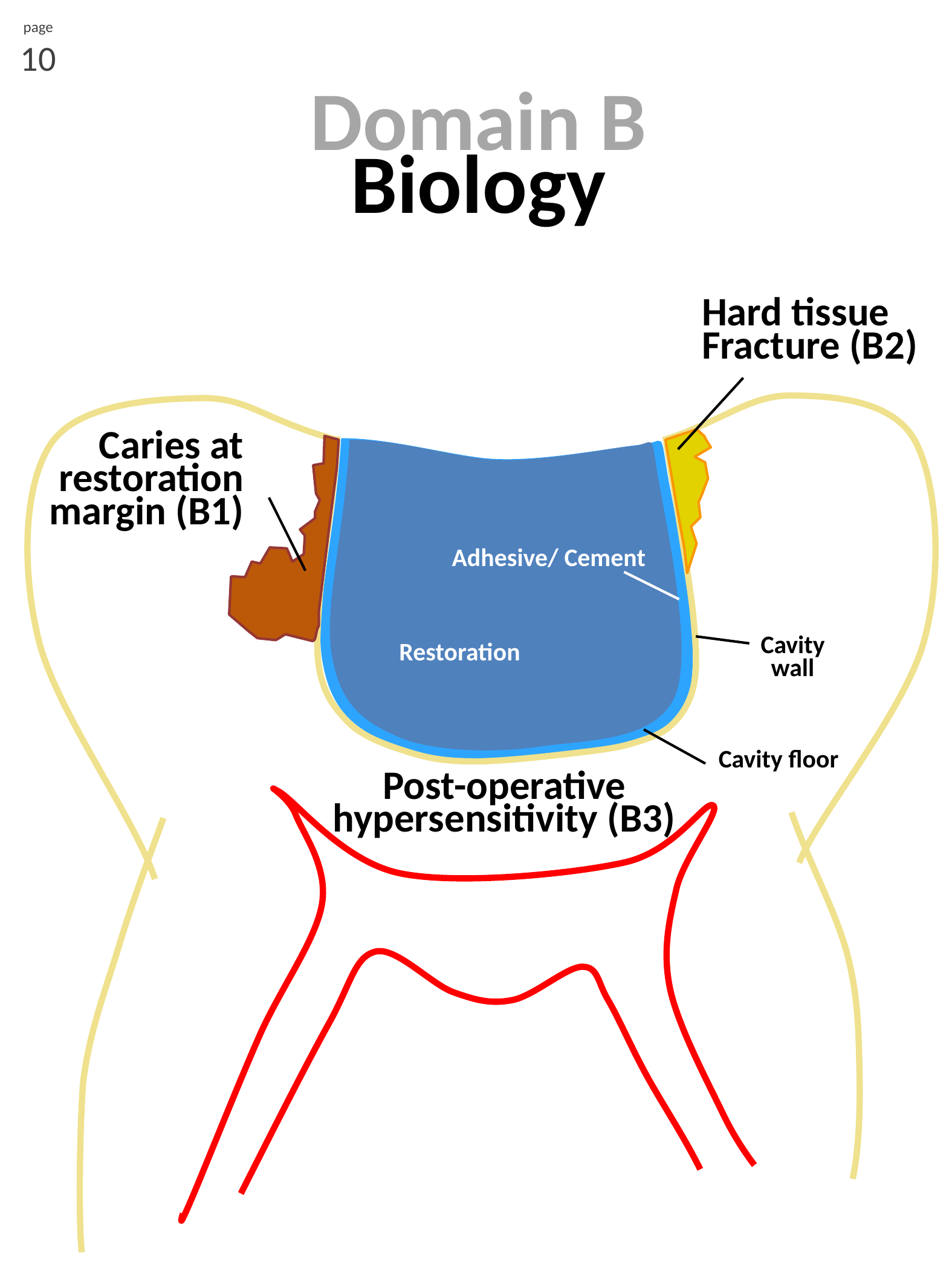

page
10
Domain B
Biology
Hard tissue
Fracture (B2)
Caries at restoration margin (B1)
Adhesive/ Cement
Restoration
Cavity wall
Cavity floor
Post-operative hypersensitivity (B3)

## Slide 11
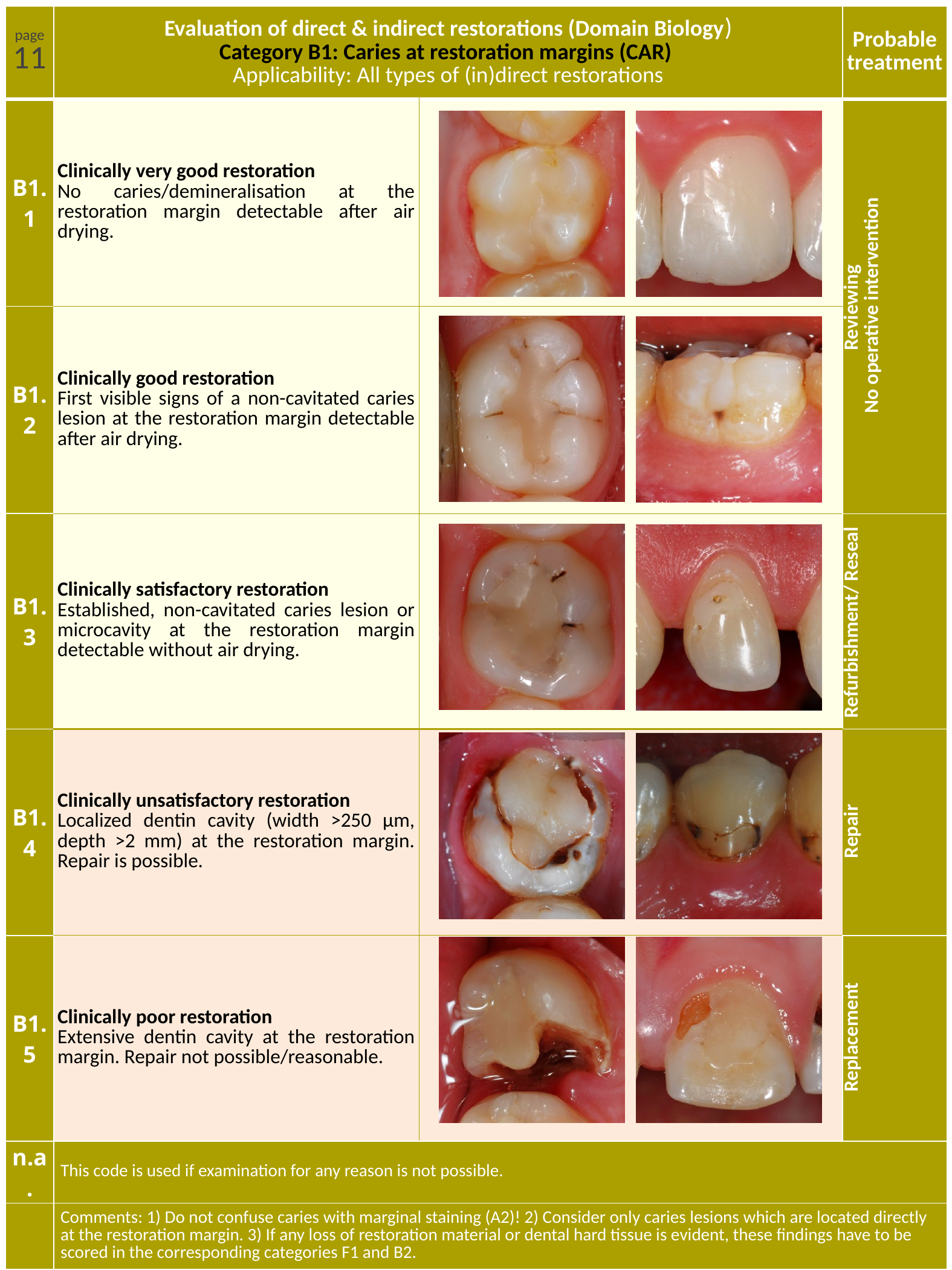

| page11 | Evaluation of direct & indirect restorations (Domain Biology) Category B1: Caries at restoration margins (CAR) Applicability: All types of (in)direct restorations | | Probable treatment |
| --- | --- | --- | --- |
| B1.1 | Clinically very good restoration No caries/demineralisation at the restoration margin detectable after air drying. | | Reviewing No operative intervention |
| B1.2 | Clinically good restoration First visible signs of a non-cavitated caries lesion at the restoration margin detectable after air drying. | | |
| B1.3 | Clinically satisfactory restoration Established, non-cavitated caries lesion or microcavity at the restoration margin detectable without air drying. | | Refurbishment/ Reseal |
| B1.4 | Clinically unsatisfactory restoration Localized dentin cavity (width >250 µm, depth >2 mm) at the restoration margin. Repair is possible. | | Repair |
| B1.5 | Clinically poor restoration Extensive dentin cavity at the restoration margin. Repair not possible/reasonable. | | Replacement |
| n.a. | This code is used if examination for any reason is not possible. | | |
| | Comments: 1) Do not confuse caries with marginal staining (A2)! 2) Consider only caries lesions which are located directly at the restoration margin. 3) If any loss of restoration material or dental hard tissue is evident, these findings have to be scored in the corresponding categories F1 and B2. | | |

## Slide 12
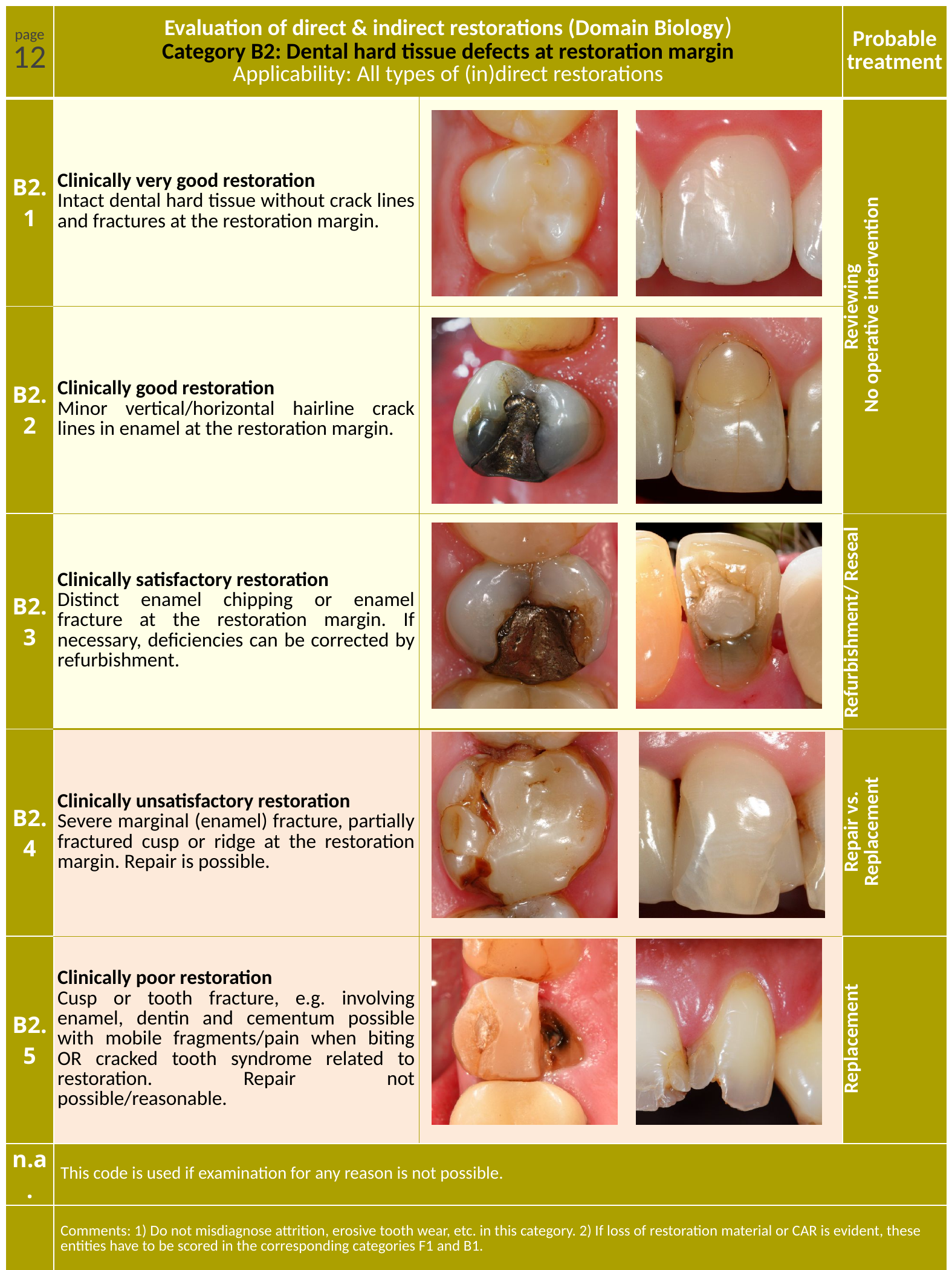

| page12 | Evaluation of direct & indirect restorations (Domain Biology) Category B2: Dental hard tissue defects at restoration margin Applicability: All types of (in)direct restorations | | Probable treatment |
| --- | --- | --- | --- |
| B2.1 | Clinically very good restoration Intact dental hard tissue without crack lines and fractures at the restoration margin. | | Reviewing No operative intervention |
| B2.2 | Clinically good restoration Minor vertical/horizontal hairline crack lines in enamel at the restoration margin. | | |
| B2.3 | Clinically satisfactory restoration Distinct enamel chipping or enamel fracture at the restoration margin. If necessary, deficiencies can be corrected by refurbishment. | | Refurbishment/ Reseal |
| B2.4 | Clinically unsatisfactory restoration Severe marginal (enamel) fracture, partially fractured cusp or ridge at the restoration margin. Repair is possible. | | Repair vs. Replacement |
| B2.5 | Clinically poor restoration Cusp or tooth fracture, e.g. involving enamel, dentin and cementum possible with mobile fragments/pain when biting OR cracked tooth syndrome related to restoration. Repair not possible/reasonable. | | Replacement |
| n.a. | This code is used if examination for any reason is not possible. | | |
| | Comments: 1) Do not misdiagnose attrition, erosive tooth wear, etc. in this category. 2) If loss of restoration material or CAR is evident, these entities have to be scored in the corresponding categories F1 and B1. | | |

## Slide 13
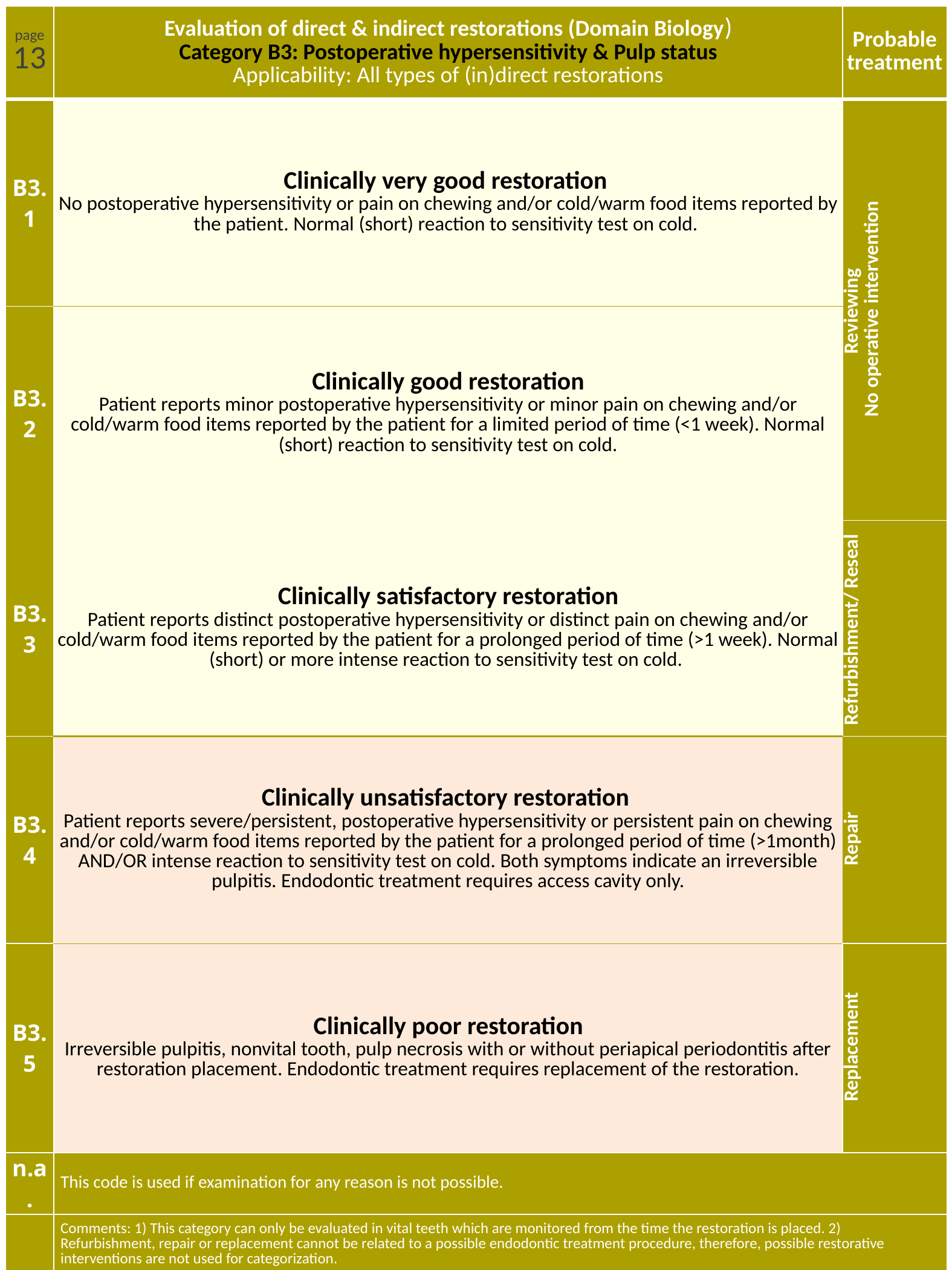

| page13 | Evaluation of direct & indirect restorations (Domain Biology) Category B3: Postoperative hypersensitivity & Pulp status Applicability: All types of (in)direct restorations | Probable treatment |
| --- | --- | --- |
| B3.1 | Clinically very good restoration No postoperative hypersensitivity or pain on chewing and/or cold/warm food items reported by the patient. Normal (short) reaction to sensitivity test on cold. | Reviewing No operative intervention |
| B3.2 | Clinically good restoration Patient reports minor postoperative hypersensitivity or minor pain on chewing and/or cold/warm food items reported by the patient for a limited period of time (<1 week). Normal (short) reaction to sensitivity test on cold. | |
| B3.3 | Clinically satisfactory restoration Patient reports distinct postoperative hypersensitivity or distinct pain on chewing and/or cold/warm food items reported by the patient for a prolonged period of time (>1 week). Normal (short) or more intense reaction to sensitivity test on cold. | |
| | | Refurbishment/ Reseal |
| B3.4 | Clinically unsatisfactory restoration Patient reports severe/persistent, postoperative hypersensitivity or persistent pain on chewing and/or cold/warm food items reported by the patient for a prolonged period of time (>1month) AND/OR intense reaction to sensitivity test on cold. Both symptoms indicate an irreversible pulpitis. Endodontic treatment requires access cavity only. | Repair |
| B3.5 | Clinically poor restoration Irreversible pulpitis, nonvital tooth, pulp necrosis with or without periapical periodontitis after restoration placement. Endodontic treatment requires replacement of the restoration. | Replacement |
| n.a. | This code is used if examination for any reason is not possible. | |
| | Comments: 1) This category can only be evaluated in vital teeth which are monitored from the time the restoration is placed. 2) Refurbishment, repair or replacement cannot be related to a possible endodontic treatment procedure, therefore, possible restorative interventions are not used for categorization. | |

## Slide 14
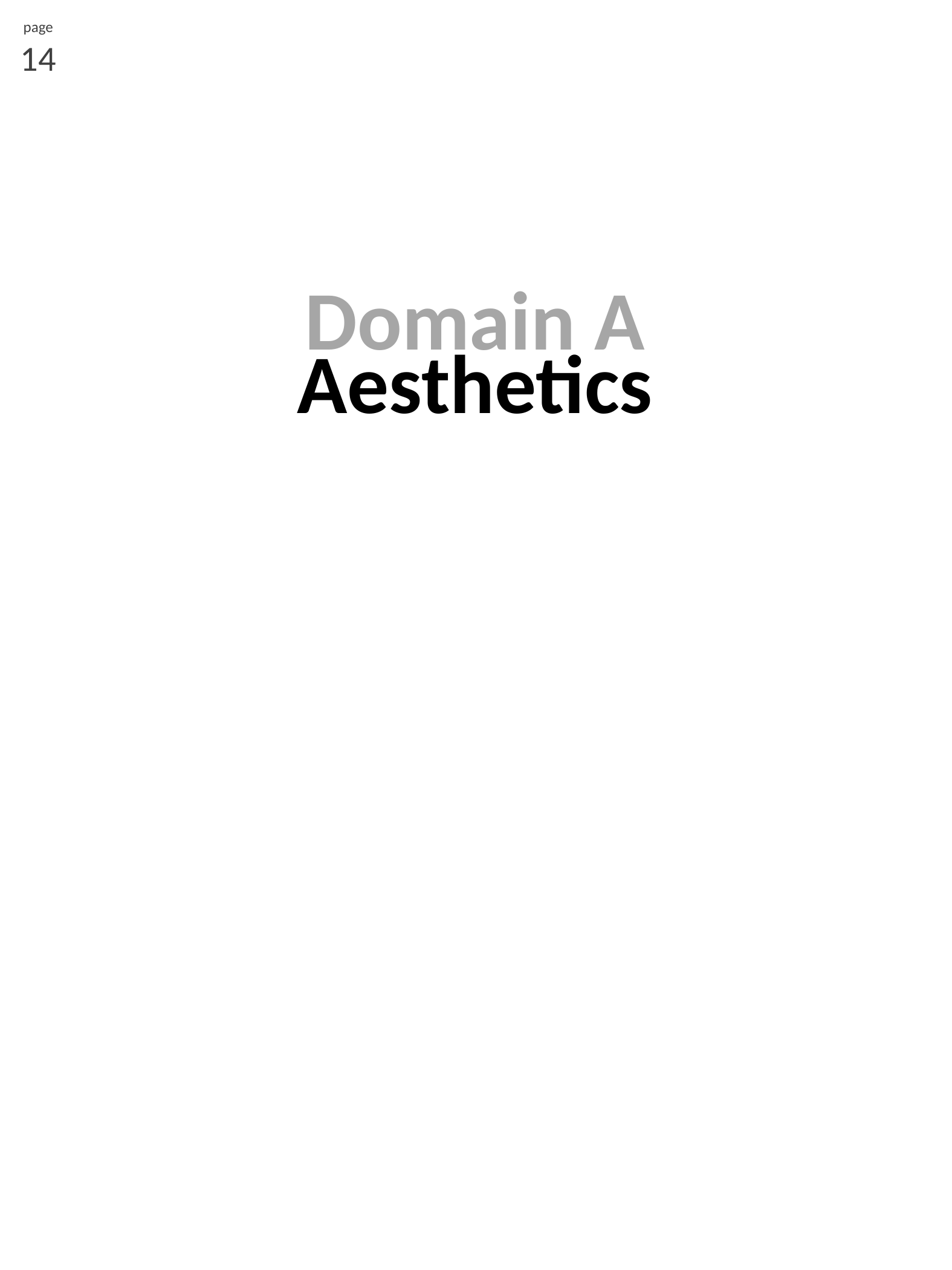

page
14
Domain A
Aesthetics

## Slide 15
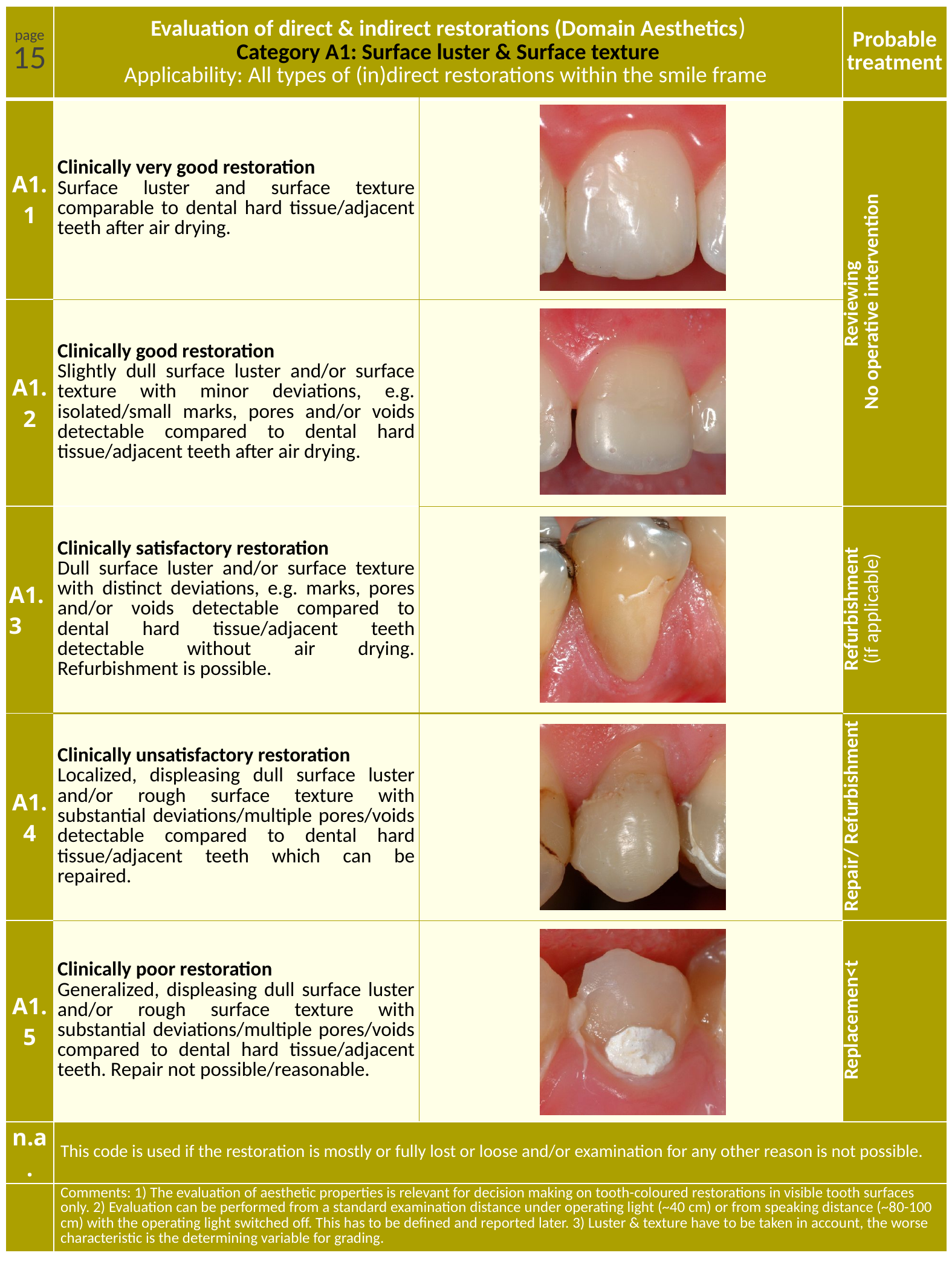

| page15 | Evaluation of direct & indirect restorations (Domain Aesthetics) Category A1: Surface luster & Surface texture Applicability: All types of (in)direct restorations within the smile frame | | Probable treatment |
| --- | --- | --- | --- |
| A1.1 | Clinically very good restoration Surface luster and surface texture comparable to dental hard tissue/adjacent teeth after air drying. | | Reviewing No operative intervention |
| A1.2 | Clinically good restoration Slightly dull surface luster and/or surface texture with minor deviations, e.g. isolated/small marks, pores and/or voids detectable compared to dental hard tissue/adjacent teeth after air drying. | | |
| A1.3 | Clinically satisfactory restoration Dull surface luster and/or surface texture with distinct deviations, e.g. marks, pores and/or voids detectable compared to dental hard tissue/adjacent teeth detectable without air drying. Refurbishment is possible. | | Refurbishment (if applicable) |
| A1.4 | Clinically unsatisfactory restoration Localized, displeasing dull surface luster and/or rough surface texture with substantial deviations/multiple pores/voids detectable compared to dental hard tissue/adjacent teeth which can be repaired. | | Repair/ Refurbishment |
| A1.5 | Clinically poor restoration Generalized, displeasing dull surface luster and/or rough surface texture with substantial deviations/multiple pores/voids compared to dental hard tissue/adjacent teeth. Repair not possible/reasonable. | | Replacemen<t |
| n.a. | This code is used if the restoration is mostly or fully lost or loose and/or examination for any other reason is not possible. | | |
| | Comments: 1) The evaluation of aesthetic properties is relevant for decision making on tooth-coloured restorations in visible tooth surfaces only. 2) Evaluation can be performed from a standard examination distance under operating light (~40 cm) or from speaking distance (~80-100 cm) with the operating light switched off. This has to be defined and reported later. 3) Luster & texture have to be taken in account, the worse characteristic is the determining variable for grading. | | |

## Slide 16
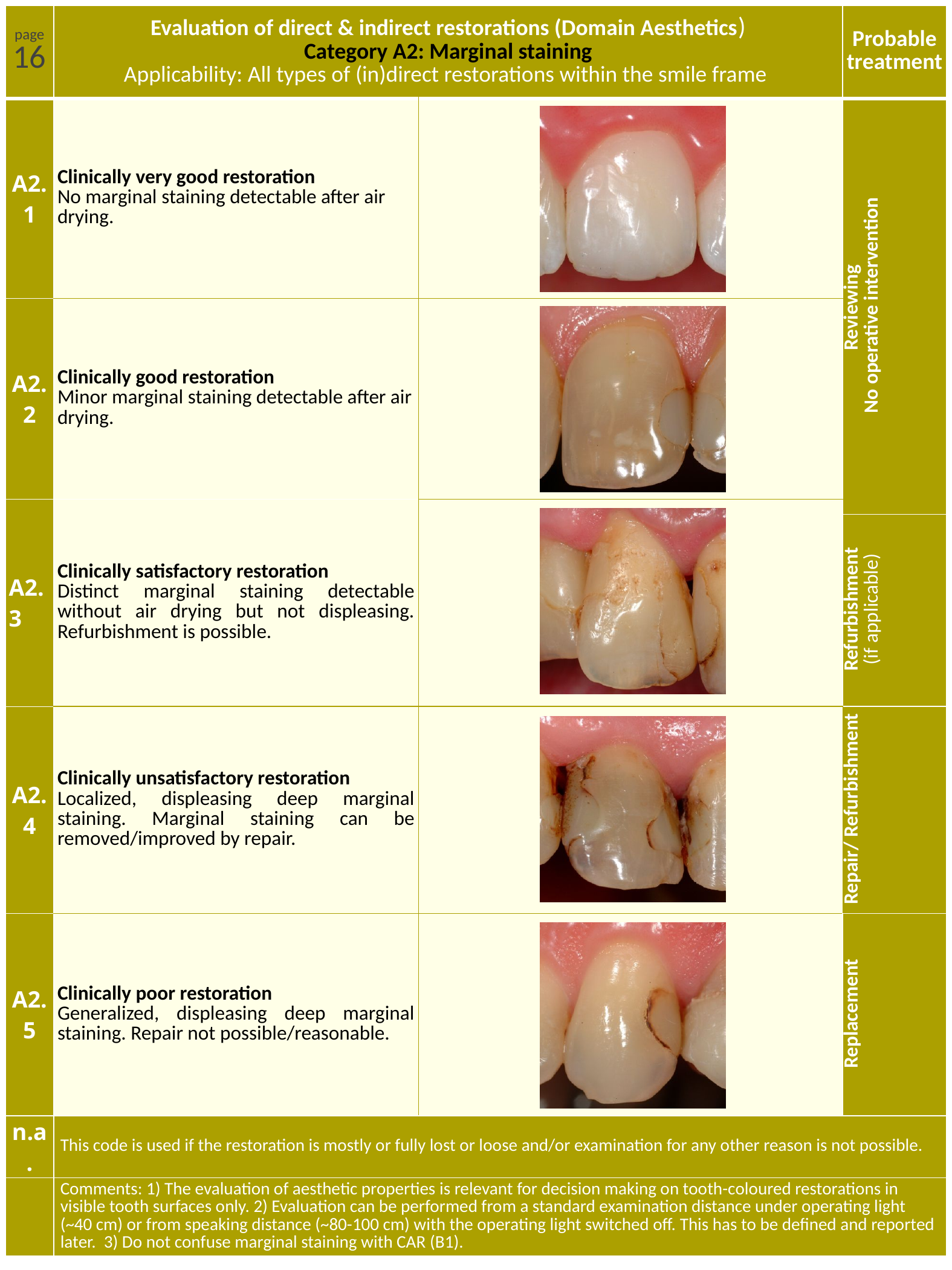

| page16 | Evaluation of direct & indirect restorations (Domain Aesthetics) Category A2: Marginal staining Applicability: All types of (in)direct restorations within the smile frame | | Probable treatment |
| --- | --- | --- | --- |
| A2.1 | Clinically very good restoration No marginal staining detectable after air drying. | | Reviewing No operative intervention |
| A2.2 | Clinically good restoration Minor marginal staining detectable after air drying. | | |
| A2.3 | Clinically satisfactory restoration Distinct marginal staining detectable without air drying but not displeasing. Refurbishment is possible. | | |
| | | | Refurbishment (if applicable) |
| A2.4 | Clinically unsatisfactory restoration Localized, displeasing deep marginal staining. Marginal staining can be removed/improved by repair. | | Repair/ Refurbishment |
| A2.5 | Clinically poor restoration Generalized, displeasing deep marginal staining. Repair not possible/reasonable. | | Replacement |
| n.a. | This code is used if the restoration is mostly or fully lost or loose and/or examination for any other reason is not possible. | | |
| | Comments: 1) The evaluation of aesthetic properties is relevant for decision making on tooth-coloured restorations in visible tooth surfaces only. 2) Evaluation can be performed from a standard examination distance under operating light (~40 cm) or from speaking distance (~80-100 cm) with the operating light switched off. This has to be defined and reported later. 3) Do not confuse marginal staining with CAR (B1). | | |

## Slide 17
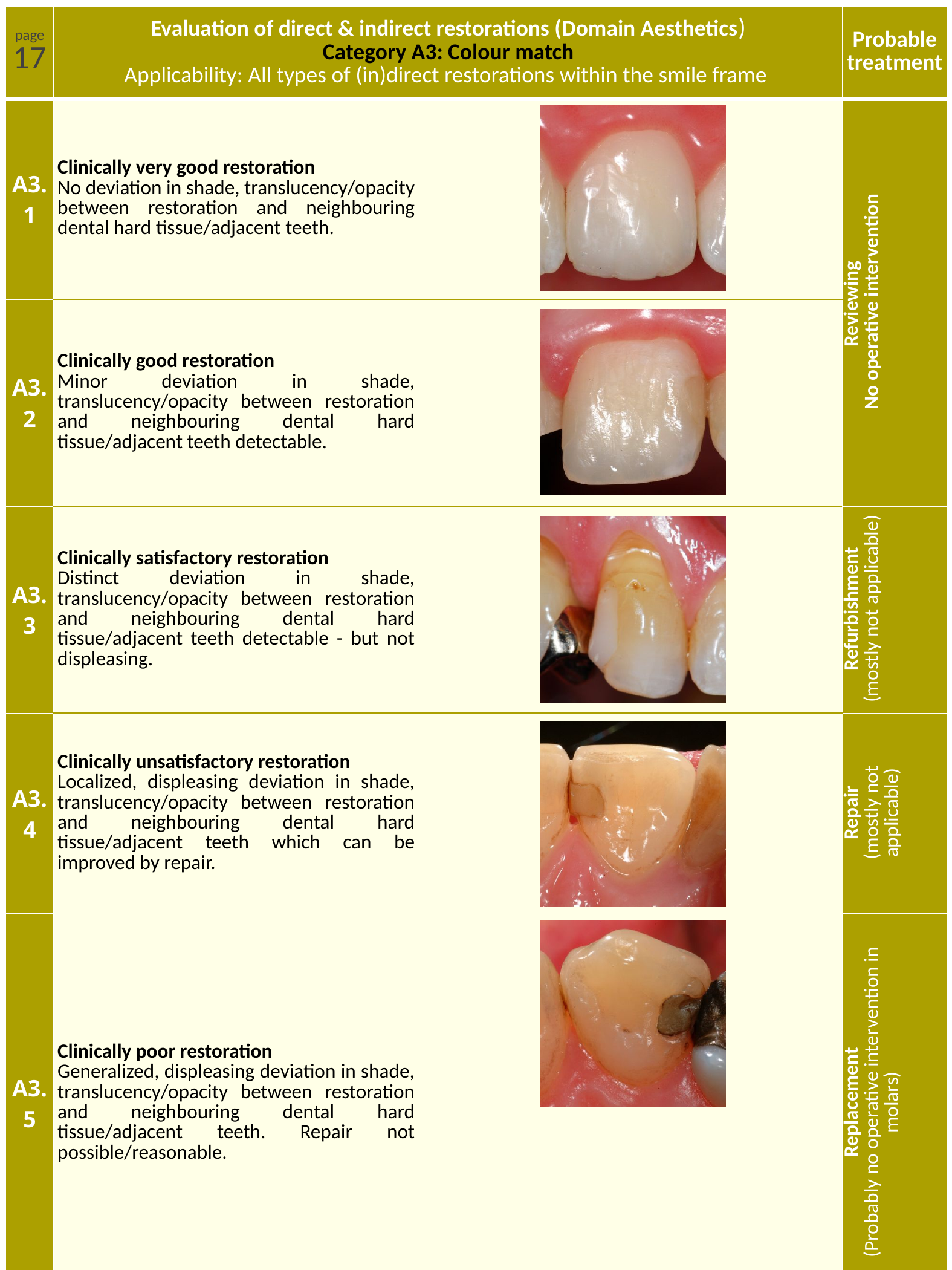

| page17 | Evaluation of direct & indirect restorations (Domain Aesthetics) Category A3: Colour match Applicability: All types of (in)direct restorations within the smile frame | | Probable treatment |
| --- | --- | --- | --- |
| A3.1 | Clinically very good restoration No deviation in shade, translucency/opacity between restoration and neighbouring dental hard tissue/adjacent teeth. | | Reviewing No operative intervention |
| A3.2 | Clinically good restoration Minor deviation in shade, translucency/opacity between restoration and neighbouring dental hard tissue/adjacent teeth detectable. | | |
| A3.3 | Clinically satisfactory restoration Distinct deviation in shade, translucency/opacity between restoration and neighbouring dental hard tissue/adjacent teeth detectable - but not displeasing. | | Refurbishment (mostly not applicable) |
| A3.4 | Clinically unsatisfactory restoration Localized, displeasing deviation in shade, translucency/opacity between restoration and neighbouring dental hard tissue/adjacent teeth which can be improved by repair. | | Repair (mostly not applicable) |
| A3.5 | Clinically poor restoration Generalized, displeasing deviation in shade, translucency/opacity between restoration and neighbouring dental hard tissue/adjacent teeth. Repair not possible/reasonable. | | Replacement (Probably no operative intervention in molars) |
| n.a. | This code is used if the restoration is mostly or fully lost or loose and/or examination for any other reason is not possible. | | |
| | Comments: 1) The evaluation of aesthetic properties is relevant for decision making on tooth-coloured restorations in visible tooth surfaces only. 2) Evaluation can be performed from a standard examination distance under operating light (~40 cm) or from speaking distance (~80-100 cm) with the operating light switched off. This has to be defined and reported later. 3) Evaluation of tooth-coloured restorations only. | | |

## Slide 18
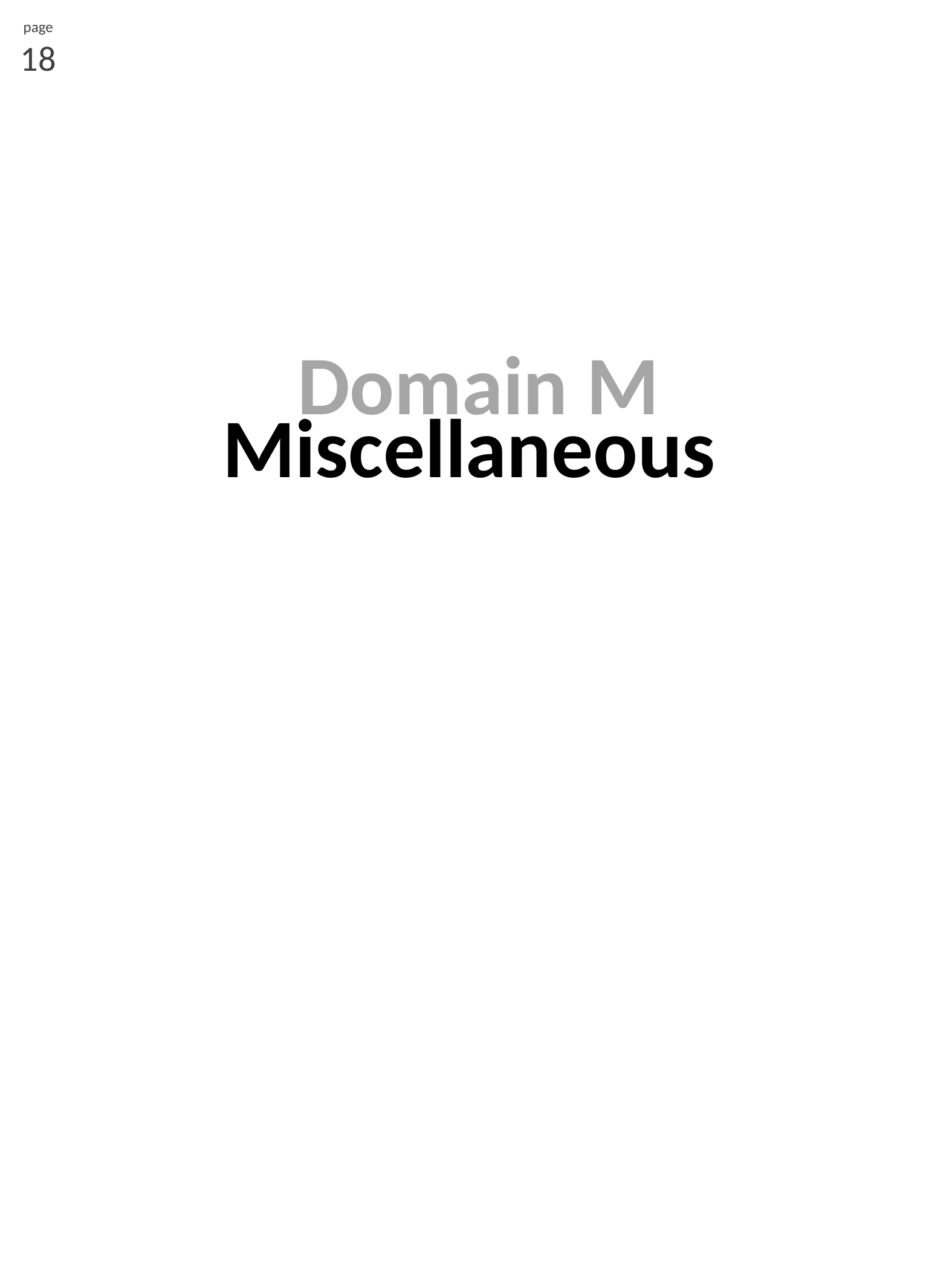

page
18
Domain M
Miscellaneous

## Slide 19
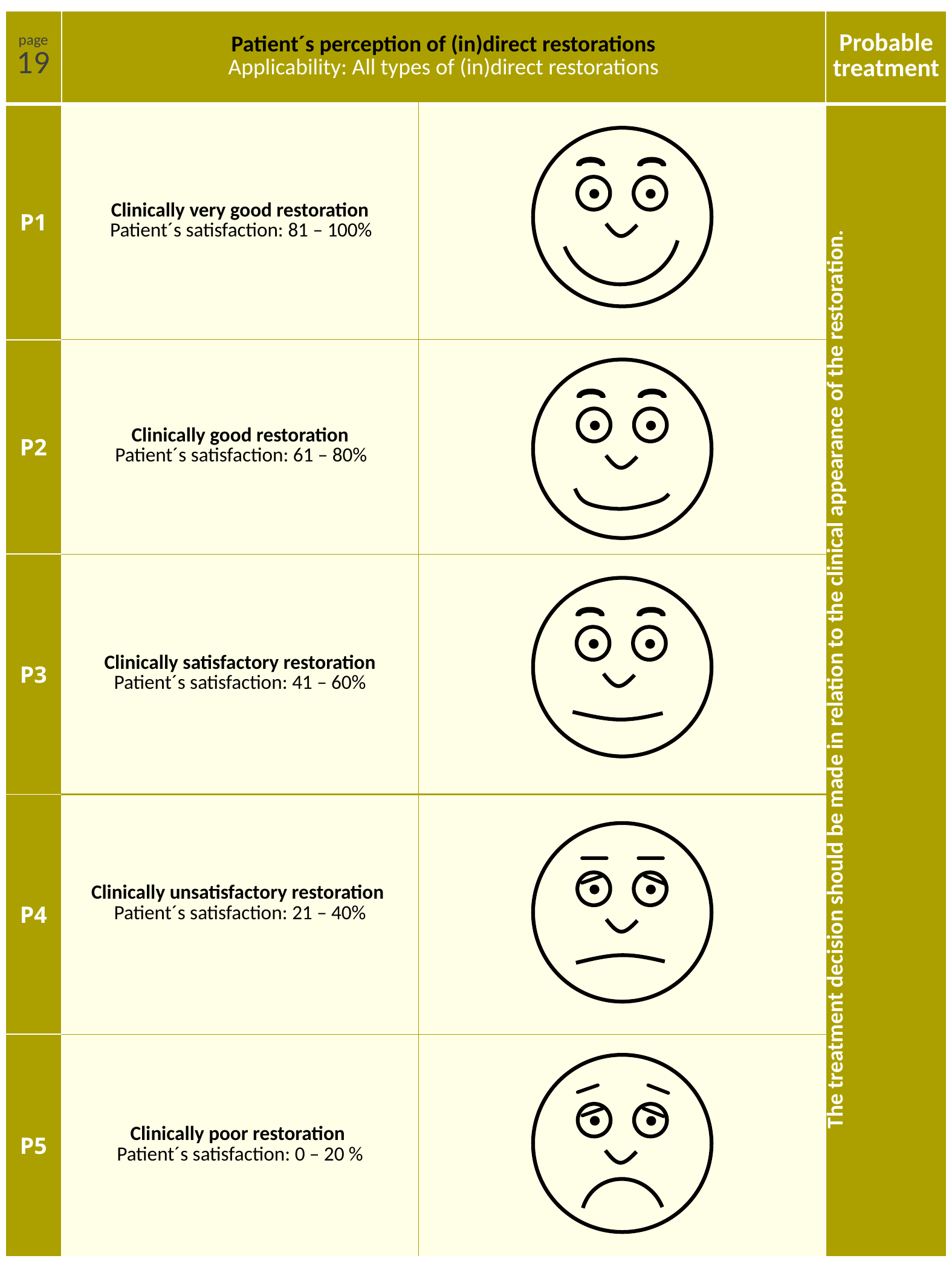

| page19 | Patient´s perception of (in)direct restorations Applicability: All types of (in)direct restorations | | Probable treatment |
| --- | --- | --- | --- |
| P1 | Clinically very good restoration Patient´s satisfaction: 81 – 100% | | The treatment decision should be made in relation to the clinical appearance of the restoration. |
| P2 | Clinically good restoration Patient´s satisfaction: 61 – 80% | | |
| P3 | Clinically satisfactory restoration Patient´s satisfaction: 41 – 60% | | |
| P4 | Clinically unsatisfactory restoration Patient´s satisfaction: 21 – 40% | | |
| P5 | Clinically poor restoration Patient´s satisfaction: 0 – 20 % | | |

## Slide 20
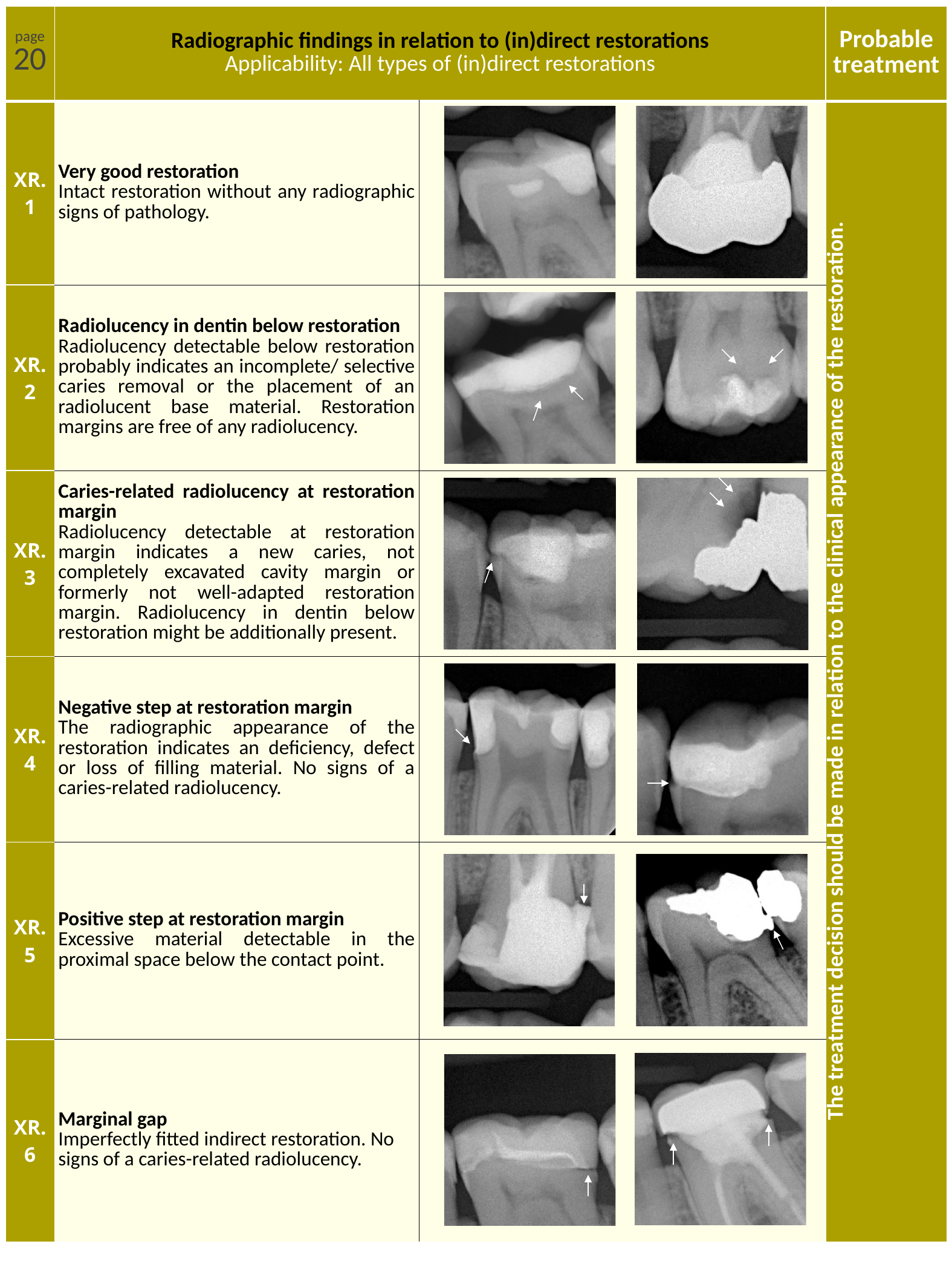

| page20 | Radiographic findings in relation to (in)direct restorations Applicability: All types of (in)direct restorations | | Probable treatment |
| --- | --- | --- | --- |
| XR.1 | Very good restoration Intact restoration without any radiographic signs of pathology. | | The treatment decision should be made in relation to the clinical appearance of the restoration. |
| XR.2 | Radiolucency in dentin below restoration Radiolucency detectable below restoration probably indicates an incomplete/ selective caries removal or the placement of an radiolucent base material. Restoration margins are free of any radiolucency. | | |
| XR.3 | Caries-related radiolucency at restoration margin Radiolucency detectable at restoration margin indicates a new caries, not completely excavated cavity margin or formerly not well-adapted restoration margin. Radiolucency in dentin below restoration might be additionally present. | | |
| XR.4 | Negative step at restoration margin The radiographic appearance of the restoration indicates an deficiency, defect or loss of filling material. No signs of a caries-related radiolucency. | | |
| XR.5 | Positive step at restoration margin Excessive material detectable in the proximal space below the contact point. | | |
| XR.6 | Marginal gap Imperfectly fitted indirect restoration. No signs of a caries-related radiolucency. | | |
